# Performance of the Longitudinal Actor–Partner Interdependence Model in Case of Large Amounts of Missing Values: Challenges and Possible Alternatives

**DOI:** 10.1017/psy.2025.18

**Published:** 2025-06-13

**Authors:** Yuanyuan Ji, Jordan Revol, Anna Schouten, Marieke J. Schreuder, Eva Ceulemans

**Affiliations:** 1 Research Group of Quantitative Psychology and Individual Differences, Faculty of Psychology and Educational Sciences, KU Leuven, Leuven, Belgium; 2 Center for Social and Cultural Psychology, Faculty of Psychology and Educational Sciences, KU Leuven, Leuven, Belgium

**Keywords:** intensive longitudinal dyadic data, longitudinal actor–partner interdependence model, missing data issues

## Abstract

Researchers interested in dyadic processes increasingly collect intensive longitudinal data (ILD), with the longitudinal actor–partner interdependence model (L-APIM) being a popular modeling approach. However, due to non-compliance and the use of conditional questions, ILD are almost always incomplete. These missing data issues become more prominent in dyadic studies, because partners often miss different measurement occasions or disagree about features that trigger conditional questions. Large amounts of missing data challenge the L-APIM’s estimation performance. Specifically, we found that non-convergence occurred when applying the L-APIM to pre-existing dyadic diary data with a lot of missing values. Using a simulation study, we systematically examined the performance of the L-APIM in dyadic ILD with missing values. Consistent with our illustrative data, we found that non-convergence often occurred in conditions with small sample sizes, while the fixed within-person actor and partner effects were well estimated when analyses did converge. Additionally, considering potential convergence failures with the L-APIM, we investigated 31 alternative models and evaluated their performance on simulated and empirical data, showing that multiple alternatives may alleviate the convergence problems. Overall, when the L-APIM fails to converge, we recommend fitting multiple alternative models to check the robustness of the results.

Intensive longitudinal designs are increasingly used to study within-dyad (e.g., mother–child, romantic partners) dynamics in daily life as well as differences in these dynamics across dyads (Bolger & Laurenceau, 2013; Iida et al., 2023). In such designs, both members of a dyad are asked to independently report on their momentary feelings, behaviors, and cognitions across multiple measurement occasions with short intervals (e.g., 24 hr or less). Usually, some of the momentary variables function as outcomes of interest, and others as predictors or covariates. For instance, Lafit et al. (2022) investigated whether the fluctuations in the affective experiences of romantic partners in daily life were predicted by one’s own and one’s partner’s enacted responsiveness. These distinct effects of one’s own covariate and that of the partner are respectively labeled actor and partner effects. Modeling such actor and partner effects requires taking into account not only dependency within *individuals*—which signifies that repeated measurements within an individual tend to exhibit similarity—but also within *dyads*—which signifies that two members within a single dyad show similarity resulting from shared experiences.

An appealing model to analyze intensive longitudinal dyadic data (dyadic ILD) and gain insight into actor and partner effects is the longitudinal version of the actor–partner interdependence model (L-APIM; Gistelinck & Loeys, 2019; Kenny et al., 2006). The L-APIM is a multilevel (i.e., measurement occasions are nested within dyads) extension of the cross-sectional actor–partner interdependence model (APIM; Kenny, 1996). The L-APIM considers not only actor and partner effects, but also accounts for remaining dependency between partners’ outcomes in the form of correlated residuals and correlated random intercepts and slopes (for recent applications of the L-APIM, see Birnie et al., 2017; Kashian, 2023; Lafit et al., 2022; Sun et al., 2017).

A major challenge when applying the L-APIM is that dyadic ILD frequently contain many missing observations. These missing data have two typical reasons. First, missing data could occur due to non-compliance that each individual member of a dyad might fail to fill out the questionnaire on one or several measurement occasions. Such non-compliance in intensive longitudinal assessments is common, as participants may become frustrated or irritated when repeatedly asked to fill out questionnaires over time (e.g., Bolger et al., 2003; Hufford, 2007), or because they simply might be unable to respond due to contextual challenges (e.g., driving a car, taking an exam, etc.). Indeed, review studies have indicated the compliance rates of intensive longitudinal studies in daily life to be around 70% to 80% (not 100%), and compliance tends to decrease further as the study progresses (e.g., Rintala et al., 2019; Wrzus & Neubauer, 2023). This can result in a fair amount of missed measurement occasions for each dyad member (Eisele et al., 2022; Silvia et al., 2013). Moreover, since dyadic studies require both members of a dyad to fill out the questionnaire to have complete data for specific measurement occasion, a measurement occasion will already be considered missing when one of the two partners do not respond, which may largely increase the number of missing measurements. Second, in some studies, missingness may also be introduced by design, such as using planned missing data to reduce participant burden and improve efficiency (Graham et al., 2006), or because the variables of interest are only reported in specific contexts that might occur infrequently, such as decision conflict (e.g., Peetz et al., 2021) and intimate partner aggression (e.g., Derrick & Testa, 2017). For instance, over the course of a 7-day study using the Experience Sampling Method (ESM) with seven measurements per day, Peetz et al. (2021) found that participants reported an average of 11.87 decision conflicts, and the variables of interest (e.g., positive or negative emotions) were only measured when participants reported experiencing a decision conflict. It is important to note that when research focuses on specific contexts, data from non-contexts may not be truly missing but simply irrelevant. However, since this also leads to a substantial reduction in the available sample size, we considered it as a form of practical missingness in this article. Moreover, missingness by design increases the likelihood of incomplete measurement occasions. Specifically, partners may provide contrasting reports about the occurrence of a specific context, leading to missing data for the variables of interest from the partner who reported no such context. This missingness could arise from various reasons, such as forgetting, unwillingness to report, or contrasting perceptions of the context. In the study by Derrick and Testa (2017), males and females, respectively, reported 178 and 264 instances of verbal intimate partner aggression by males, but only 34 instances matched between partners. Taken together, if one or both of the missing data issues described above occur in dyadic ILD, this might amount to large losses of data, as is illustrated in Figure 1.

**Figure 1:**
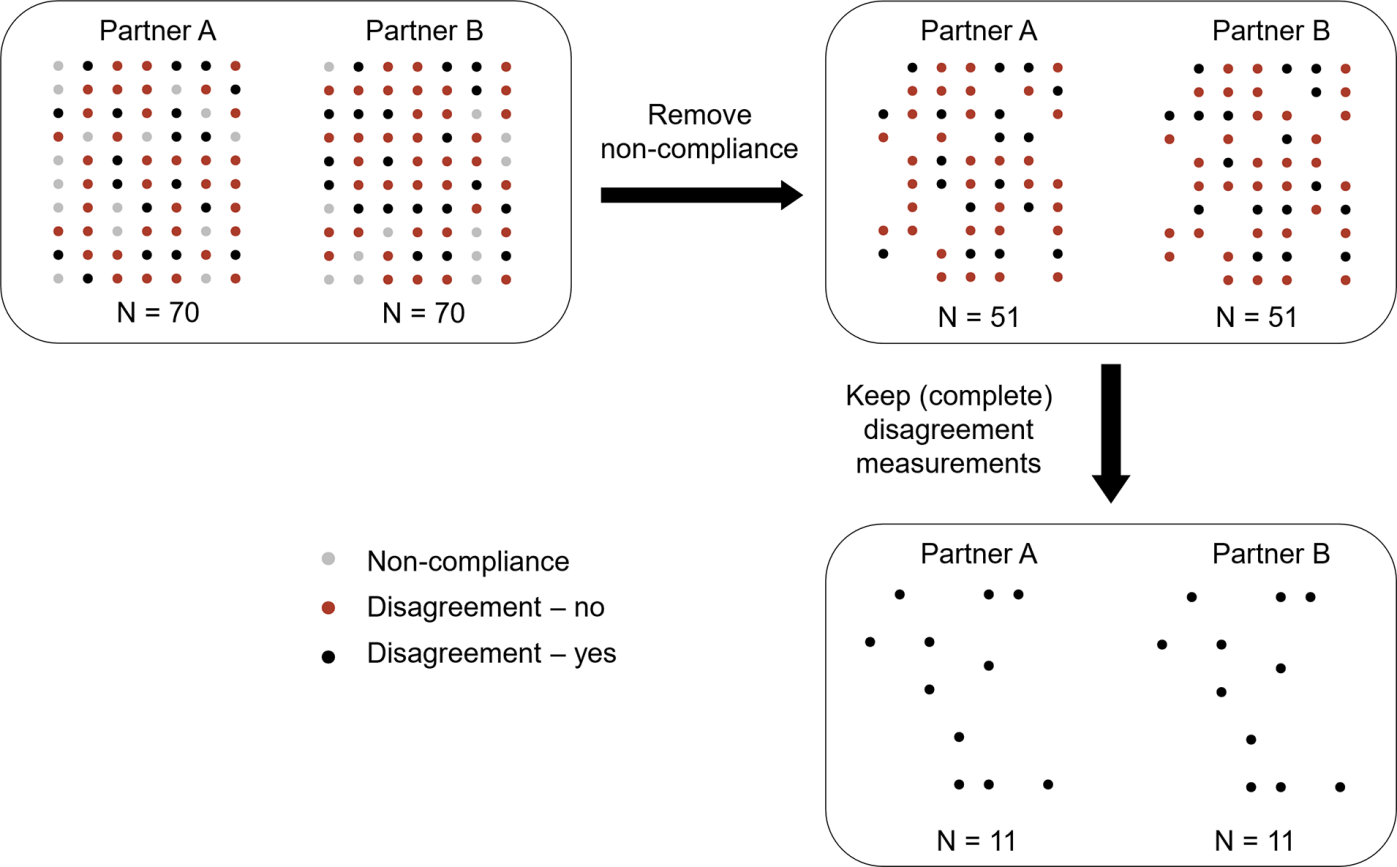
Graphic representation of missingness issues through a simulated example. *Note*: The intended number of measurement occasions per partner is 70. The reported measurements are represented by dots, with black ones indicating the occurrence of couple disagreement. The compliance rate for each partner is approximately 80%, the probability of couple disagreement is 33%, and partners provide non-matching reports around 50% of the time. Only 11 complete measurements remain for the dyad as a whole, indicating a great extent of data loss.

Large amounts of missing data might lead to a variety of statistical challenges when fitting the L-APIM. Specifically, as we will demonstrate using an empirical daily diary dataset on couple disagreement, it might lead to non-convergence issues. Hence, the first aim of this article is to systematically examine the performance of the L-APIM. To achieve this goal, we will perform Monte Carlo simulations in which we will generate datasets based on the characteristics of the daily diary data, while manipulating the amount of missing data. We will evaluate the performance of L-APIM by inspecting convergence, bias and uncertainty of the estimates. These manipulations will provide insight into when convergence issues become relevant and how the estimation of L-APIM is influenced.

Additionally, we aim to provide insights into alternative models that can be considered when the L-APIM fails to perform well. To this end, we will first introduce simpler models by 1) restricting the variance–covariance structures of residuals and random effects as demonstrated by Gistelinck and Loeys (2019) and 2) considering frequently used strategies in empirical research, such as focusing solely on the actor effect while disregarding the partner effect (e.g., Hsueh et al., 2018). The estimation accuracy of these alternative models remains unclear, as they disregard some features of the true underlying data generating process, which might lead to bias or increased estimation uncertainty. Therefore, our second goal is to explore the estimation performance of these alternative models by fitting them to simulated datasets for which the L-APIM failed to converge and evaluating their performance in terms of the same three criteria (convergence, bias, and uncertainty of the estimates). By doing so, we aim to give suggestions on which modeling approach might still be feasible (if any) when faced with extensive missing data issues in intensive longitudinal dyadic studies. Finally, we will apply the alternative models to the illustrative daily diary data to examine if the insights gained from the simulation study hold true in practical applications.

This article is structured as follows: The first section focuses on the L-APIM. Specifically, we will discuss the data structure of dyadic ILD and the L-APIM in detail, and we will assess the performance of the L-APIM on the empirical daily diary data, and through a simulation study. The second section shifts its focus to alternative models. It commences with an overview of all alternative models under consideration, and then evaluates their performance on simulated datasets. Finally, we examine the generalizability of our simulation findings by applying promising alternative models to the daily diary data. We will conclude with a general discussion of our findings and additional considerations.

## 1 Testing the performance of L-APIM

### 1.1 Dyadic ILD: daily diary dataset

We demonstrate dyadic ILD using a 14-day diary dataset collected by Schouten (2022) in 75 Belgian heterosexual romantic couples (*M*_age_ = 31.75, *SD*_age_ = 10.66). She aimed to investigate whether romantic partners’ emotions and behaviors during disagreements can be predicted by the extent to which they (actor effect) and their partners (partner effect) achieved specific relationship goals. At the end of each day, participants were instructed to reflect on whether they had experienced a disagreement with their partner. If they had a disagreement, participants were asked to report on 18 emotions (e.g., “ashamed,” “annoyed”), 15 behaviors (e.g., “I listened to my partner’s point of view”), and achievement of 15 relationship goals (e.g., “To what extent were you able to act and think freely?”) during the disagreement using seven-point Likert scales. Per disagreement, the scores were combined into four emotion scales (Negative disengaging, Negative engaging, Positive, and Worry), two behavior scales (Considerate and Evasive), and three goal scales (Autonomy, Relatedness, and Social roles) (see Supplementary Material).

**Figure 2:**
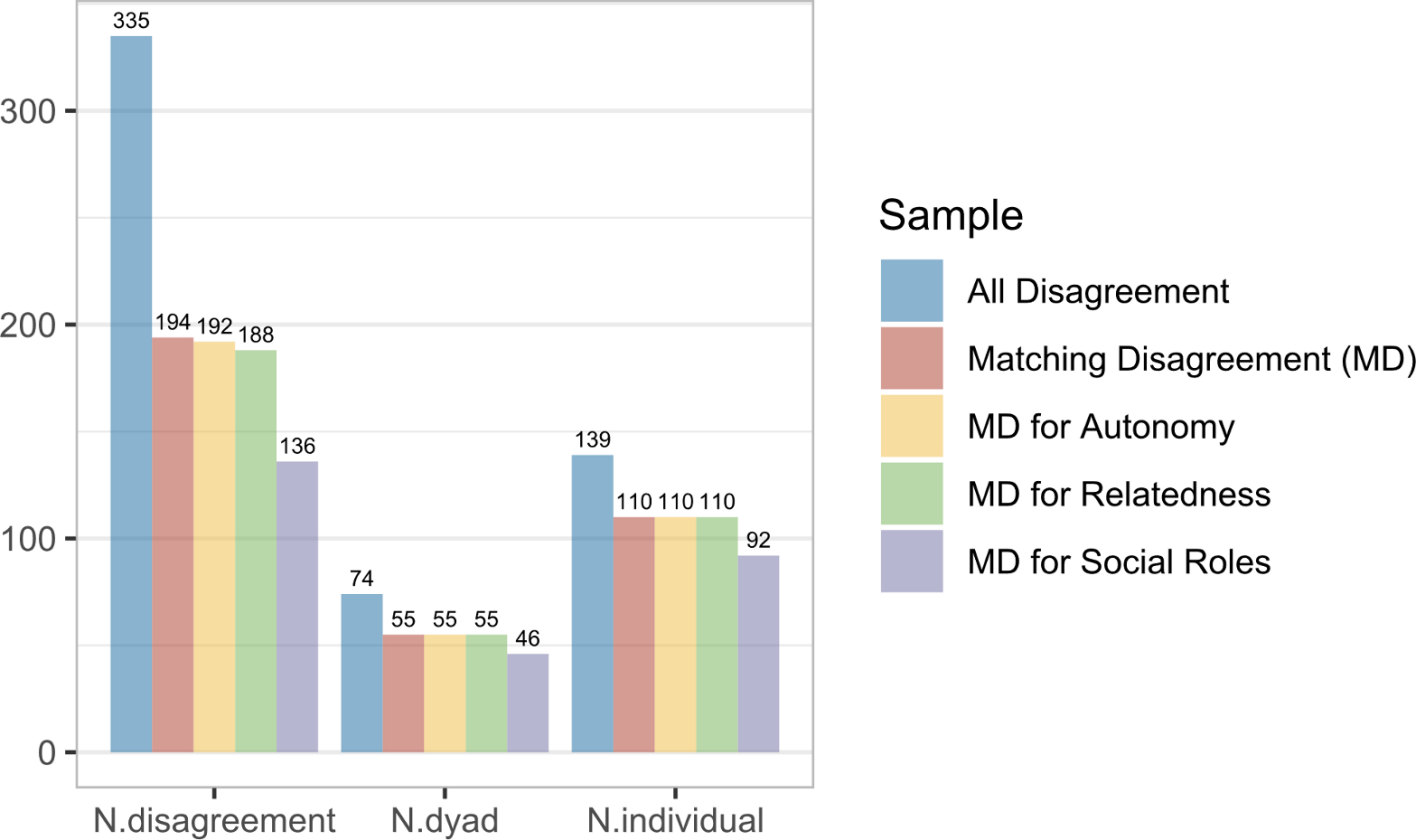
Number of measurements that correspond to a disagreement in the daily diary dataset. *Note*: “N.disagreement” is the number of measurements that correspond to a disagreement, “N.dyad” and “N.individual” are the number of dyads and individuals that provided at least one disagreement report.

The daily diary data contain many missing values because of three reasons. The daily diary data contain many missing values because of three reasons. First, participants only reported their emotions and relationship goals on days where disagreements occurred (with an average of 2.23 disagreement days). Second, partners often did not agree about the occurrence of a disagreement, leading to measurements of emotions and relationship goals being available for one of the two dyadic members only. Specifically, both partners indicated that a disagreement occurred on only 1.29 days on average, yielding little matching reports. Third, participants only reported on the achievement of relationship goals when they had indicated that that goal was important during that disagreement. This further reduces the amount of available matching reports to 192 for autonomy, 188 for relatedness, and 136 for social roles. Figure 2 provides further descriptive information.

Table 1 shows the data structure of the daily diary dataset. We present the data in the “long stacked format,”^1^ where measurements from a couple on one day span two rows, one per participant, and each person’s predictor score serves to predict both their own outcome (actor effect) and their partner’s (partner effect, indicated by the *.p* notation of the predictor score). The first to third columns of the table contain indices that will be used throughout the article, including dyad.ID (*i* = 1,…, *N*), partner within dyad (*j* = F or M, representing female or male, respectively), and measurement day within dyad (*k* = 1,…, *K_i_*, where *K_i_* is the number of measurements occasions within dyad *i*). The “Missing” column indicates whether the measurement is missing for a specific partner. The 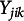 column is the outcome variable (e.g., positive emotion).

**Table 1:** Example rows of intensive longitudinal dyadic dataset

Dyad.	Partner	*Measurement*		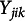				
ID (*i*)	within	day within		[Positive	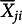	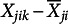	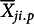	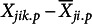
	dyad (*j*)	dyad (*k*)	Missing	emotion]	[Autonomy]	[Autonomy]	[Autonomy]	[Autonomy]
1	F	1	1	–	–	–	3.66	−0.24
1	M	1	1	1.28	3.66	−0.24	–	–
1	F	2	0	1.85	4.03	0.28	3.66	−0.13
1	M	2	0	0.83	3.66	−0.13	4.03	0.28
1	F	3	0	3.65	4.03	−0.33	3.66	0.27
1	M	3	0	1.8	3.66	0.27	4.03	−0.33
…	…	…	…	…		…		…
2	F	1	1	2.55	2.87	0.02	–	–
2	M	1	1	–	–	–	2.87	0.02
2	F	2	1	–	–	–	–	–
2	M	2	1	–	–	–	–	–
2	F	3	0	0.79	2.87	−0.34	4.57	−0.01
2	M	3	0	3.22	4.57	−0.01	2.87	−0.34
…	…	…	…	…	…	…	…	…

*Note*: In the “Missing” column, “1” indicates that the measurement is considered missing and is excluded from estimation, “0” indicates that the measurement is non-missing and thus included in estimation.

As suggested by Gistelinck and Loeys (2019), we disentangled the time-varying predictors (e.g., autonomy) of each partner into two components: a time-average ( 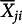 ) and a time-specific ( 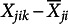 ) component to capture both between- and within-person effects. Specifically, 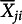 is the average value of partner *j* within dyad *i.* Note that we could also have opted to use the dyad averages rather than the partner averages in this step. We did not do this because we focused on distinguishable dyads (i.e., distinguishing between men and women) and aimed to estimate the effects for both partners separately.

### 1.2 L-APIM

#### 1.2.1 Model

L-APIM is a multilevel model for dyadic ILD, such as the daily diary data described previously. In this model, the outcome scores of partner *j* (*j* = F or M) at the *k*th measurement of the *i*th dyad ( 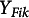 and 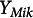 ) are predicted based on their own time-average (resp. 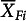 or 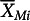 ) and time-specific (resp. 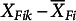 or 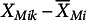 ) predictor scores, yielding the between-person ( 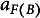 and 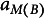 ) and within-person ( 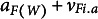 and 
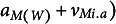
 actor effects, and those of their partner, implying the between-person ( 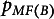 and 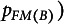 and within-person ( 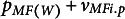 and 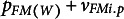 ) partner effects. The L-APIM is formulated as follows:
(1)

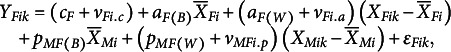



(2)

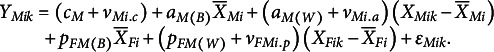



The fixed intercepts ( 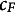 and 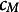 ) of the two partners are allowed to differ. Random effects for each intercept ( 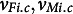 ) and within-person actor ( 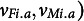 and partner slopes ( 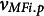 , 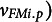 are also allowed to differ between the two partners. To account for the interdependence of dyadic partners, these random effects are assumed to be multivariate normally distributed:
(3)

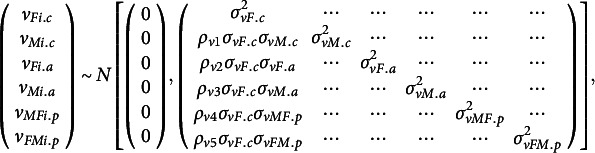

where 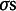 indicate the standard deviations of each random effect and 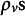 indicate the correlations between specific pairs of random effects, for example, 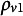 represents the correlation between the random intercepts for the female 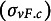 and male 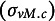 partners. Additionally, the level 1 residuals ( 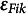 and 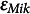 ) are assumed to be bivariate normally distributed:
(4)

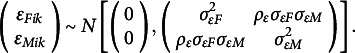



The error variance is allowed to differ across the partners. The correlation 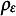 again captures dyadic interdependence, but now at the level of the measurement occasions. Note that we did not consider temporal dependencies such as autoregressive effects between measurements, because the use of conditional questions and a large amount of missing data hinders proper estimation of such dependencies.

#### 1.2.2 Estimation

We used measurement-specific pairwise deletion to handle missing data. This means that we excluded all measurements for which one or more variables of one or both partners were missing. Since measurements from a dyad at a specific time point span two rows in the “long stacked format,” both rows were removed from estimation when mismatching reports occurred. All analyses were conducted in R (R Core Team, 2021). The full set of L-APIM parameters can be estimated using the SEM framework (e.g., Gistelinck & Loeys, 2019) or multilevel modeling (e.g., Lafit et al., 2021). We adopted the latter approach because implementing L-APIM with random slopes is relatively challenging using the “lavaan” package in R (Rosseel, 2012). A preliminary simulation study was conducted to compare the performance of different estimation methods for multilevel modeling, including Bayesian estimation using the “brms” package (Bürkner, 2017) and frequentist estimation using the “lme()” function and restricted maximum likelihood estimation method (REML) in the “nlme” package (Pinheiro et al., 2023), with two separate optimizers, “optim” and “nlminb.” The “optim” showed the best convergence as well as short computation time (see details in Supplementary Material). Therefore, we focus on the optimizer “optim” in the remainder of this article. The code to do this can be found at https://osf.io/698te/.

### 1.3 L-APIM results for the daily diary data

We fit multiple L-APIM models to the daily diary data, each time using one of the three types of relationship goals (autonomy, relatedness, and social roles) as predictor, and one of the six emotions and behaviors (negative disengaging emotion, negative engaging emotion, positive emotion, worry, considerate behavior, and evasive behavior) as outcome. Considering all possible predictor–outcome combinations, we fitted a total of 18 L-APIMs. Table 2 reports whether the different models converged and, if so, provides the estimates of the between-person and fixed within-person actor and partner effects.

**Table 2:** L-APIM results for the empirical daily diary data

Predictor	Outcome	Converge?	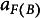	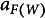	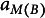	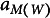	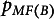	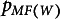	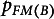	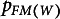
Autonomy	ND	Yes	-0.57^**^	-0.55	-0.14	-0.67^***^	0.33	-0.27	-0.09	-0.27
	NE	Yes	-0.26	0.10	-0.26	0.01	0.35^*^	-0.04	0.00	-0.62^*^
	PA	Yes	0.30^*^	0.32	0.36^*^	0.04	-0.20	0.08	-0.04	0.28
	WA	No	-	-	-	-	-	-	-	-
	CB	Yes	0.31	0.30	0.29	0.71^***^	0.05	0.15	0.08	0.15
	EB	No	-	-	-	-	-	-	-	-
Relatedness	ND	No	-	-	-	-	-	-	-	-
	NE	No	-	-	-	-	-	-	-	-
	PA	No	-	-	-	-	-	-	-	-
	WA	Yes	0.14	-0.16	0.47^**^	0.26	-0.09	-0.16	0.05	0.04
	CB	Yes	0.38^**^	0.30	0.21	0.65^***^	0.09	-0.15	0.10	0.04
	EB	Yes	-0.23^*^	-0.15	-0.22^*^	-0.31^*^	0.03	-0.14	-0.02	-0.04
Social roles	ND	Yes	-0.50^**^	-0.47^*^	-0.03	-0.38	0.26	-0.03	-0.27	-0.42^*^
	NE	No	-	-	-	-	-	-	-	-
	PA	No	-	-	-	-	-	-	-	-
	WA	Yes	0.00	-0.66^**^	0.23	-0.30	-0.13	0.29	-0.20	0.09
	CB	Yes	0.42^**^	0.18	0.49^***^	0.49^*^	0.11	-0.14	-0.01	0.20
	EB	No	-	-	-	-	-	-	-	-

*Note*: ND, Negative Disengaging emotion; NE, Negative Engaging emotion; PA, Positive emotion; WA, Worry; CB, Considerate Behavior; EB, Evasive Behavior. ***p < .001, **p < .01, *p < .05.

It is important to note that statistical issues arose when fitting the L-APIMs. Specifically, among the 18 L-APIMs examined, 8 failed to converge, implying that no estimates of the actor and partner effects were obtained. In the other 10 models, several effects were found to be significantly different from zero. Regarding actor effects, some results were consistent across genders. For example, both females and males exhibited more positive emotions when their own autonomy goals were generally (i.e., between-person actor effect) fulfilled to a higher extent. On the other hand, some actor effects varied across partners, for instance, daily (i.e., within-person) achievement of the autonomy goal predicted reduced negative disengaging behavior in men but not in women. Regarding partner effects, we found that most of these effects were not significant. This is in line with previous work showing that when self-report measures are used, partner effects are typically smaller than actor effects (Orth, 2013). An example of a significant partner effect is found in the positive association between the male’s average level of autonomy fulfillment and the female’s negative engaging emotions.

### 1.4 L-APIM results for simulated datasets

While non-convergence issues were observed for the daily diary dataset, the underlying reasons remained unclear. In addition, the estimation reliability was unknown due to the absence of the “true value” in empirical dataset. Therefore, we conducted Monte Carlo simulations to systematically evaluate L-APIM performance in terms of its convergence, estimation bias, and uncertainty across multiple conditions. In the simulation, we mainly adopted the missingness by design perspective, where measurements that occurred outside a specific context (e.g., disagreement) are considered irrelevant and hence are removed. Moreover, additional missingness could occur in some conditions due to partners disagreeing about the occurrence of the context.

#### 1.4.1 Simulation design

Data were generated by adapting the L-APIM simulation function from Lafit et al. (2021). We sampled the time-specific and time-average predictor scores of both partners as well as their level 1 errors and random effects from multivariate normal distributions with means and (co)variances as specified in Table 3. Using the L-APIM equations, these predictor scores and level 1 errors were combined into the outcome scores. The parameters for data generation were derived from one of the L-APIM model in the daily diary data analysis (see Table 3, where “autonomy” was selected as the predictor and “negative disengaging emotion” as the outcome). To mimic real-life scenarios, we assumed that the context of interest occurred in 10% of all potential measurement occasions. The following factors were manipulated in the simulation:

- Number of dyads (*N*) with two conditions: 50, 100 (yielding, respectively, 100 or 200 participants). The number of dyads was chosen based on frequently occurring numbers in research (e.g., Pesch et al., 2018; Zeijen et al., 2020).
- Number of potential measurement occasions per dyad (*K*) with two conditions: 14, 70. These numbers correspond to commonly used numbers in experience sampling (ESM) studies (70 measurements in total) and daily diary studies (14 days).
- Context-specific data were sampled by randomly selecting 10% of all potential measurement occasions under two conditions: completely matching and partially matching. In the completely matching condition, the same measurement days are kept as context-specific for both partners (i.e., they perfectly agreed about the occurrence of a disagreement). In contrast, the partially matching condition allowed for incongruence between two partners’ reports of the context, which leads to additional missingness. Meanwhile, we also considered the fact that partners are likely to agree on the occurrence of a context to some degree. To achieve this, we intentionally set 20% of the context-specific data to match between partners, while the remaining 80% was independently sampled, regardless of dyadic relationship, meaning that missingness due to contrasting reports of contexts occurred completely at random (MCAR). We chose these two conditions to represent cases where partners’ reports were completely identical or largely independent from each other and thus non-overlapping.

**Table 3:** Parameters for data generation

Parameter	Value for data generation						
Number of dyads (*N*)	[50, 100] (75)						
Number of measurements per dyad (*K*)	[14, 70] (14)						
Missing percentage	90% (90.76%)						
Fixed effects		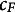	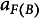	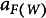	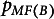	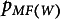	
		3.9	−0.6	−0.6	0.3	−0.3	
		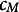	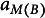	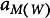	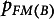	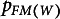	
		3.3	−0.1	−0.7	−0.1	−0.3	
Grand mean for predictor		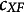	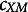				
		4.5	4.5				
SD and correlation of		*SD*	correlation				
Residuals	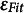	0.95	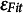				
	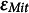	1.05	0.30				
Random effects	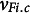	1.01	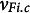	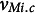	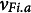	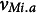	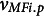
	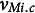	0.43	−0.35				
	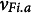	1.13	−0.01	−0.25			
	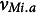	0.26	0.89	−0.33	−0.29		
	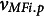	0.49	−0.24	0.23	−0.95	0.10	
	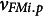	0.44	0.08	−0.32	0.97	−0.23	−0.94
Time-specific part for predictor	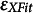	0.8	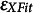				
	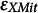	0.9	−0.1				
Time-average part for predictor	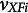	0.7	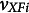				
	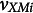	0.6	0.6				

*Note:* Numbers in the brackets for the first three rows indicate the parameter values from the empirical data. The other parameter values used for data generation were directly derived from the empirical data.

Overall, there were eight conditions. For each condition, we conducted 1,000 replicates, yielding 8 × 1,000 = 8,000 datasets.

#### 1.4.2 Simulation analysis

An L-APIM was fitted to each of the 8,000 datasets after pairwise deletion of the missing data. We will report the following results^2^:

- The number of measurements included in model estimation.
- The proportion of non-convergence across Monte Carlo replicates.
- Relative bias, computed as the difference between the estimated effect for each replicate and the true value, divided by the true value. As suggested in previous research (Muthén & Muthén, 2002), we considered the acceptable relative bias to be 10%, implying, for instance, a bias of 0.05 for true effects equal to 0.5.
- Standard error (SE) to assess estimation uncertainty, computed as the standard deviation of the estimated fixed effects across the converged replicates divided by the square root of the total number of converged replicates. Larger SE values indicate higher uncertainty.

#### 1.4.3 Simulation results

*Number of measurements included in model estimation*. As displayed in Table 4, under partially matching conditions, fitting the L-APIM led to a substantial reduction in available sample size.

**Table 4:** Number of measurements included in estimation and number of converged replicates

Matching pattern	*N* × *K*	Number of measurements *M(SD)*	Number of converged replicates
Completely matching	50 × 14	140 (0)	270
	100 × 14	280 (0)	411
	50 × 70	700 (0)	807
	100 × 70	1,400 (0)	945
Partially matching	50 × 14	41.40 (4.77)	13
	100 × 14	83.12 (6.51)	16
	50 × 70	208.35 (10.47)	560
	100 × 70	417.59 (15.08)	699

*Note*: The total number of potential measurements can be calculated as *N* (number of dyads) × 2 (two partners within a dyad) × *K* (number of measurement days per dyad).

*Convergence*. All conditions exhibited convergence rates lower than 90%, except for the 100 × 70 condition, which implies the largest sample size (see Table 4). Extremely low convergence rates (<10%) were observed in partially matching conditions with small sample size (*K* = 14).

**Figure 3:**
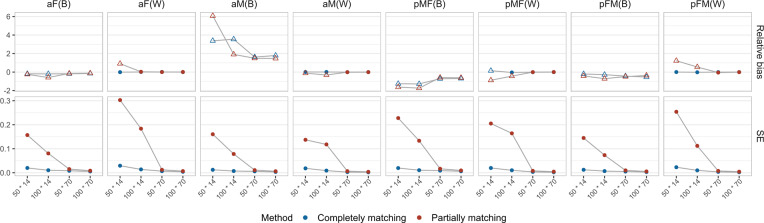
Relative bias and SE of the L-APIM estimates as a function of the manipulated factors. *Note*: The *x*-axis indicates *N* (number of dyads) * *K* (number of measurement day per dyad). The upper panels display the average relative bias across converged replicates for each estimate, while the lower panels show the standard error (SE). The open dots on the upper panels represent biases larger than 10%.

*Relative bias and SE*. Figure 3 shows the average relative bias and SE across converged replicates. Regarding relative bias, larger biases were observed for the between-person estimates, all exceeding 10%. These biases tended to decrease as the sample size increased. For within-person estimates, most biases remained within the acceptable 10% range, though biases greater than 10% were observed in case of smaller number of measurement occasions per dyad (*K* = 14) under partially matching conditions.

In terms of SE, we found a consistent trend where the SE decreased with an increase in sample size, indicating less uncertainty. Additionally, completely matching conditions generally had smaller SE values than partially matching conditions, especially when *K* = 14, which is also due to differences in sample size. It is noteworthy that when *K* = 14, the SE values were sometimes larger than 0.2, with the underlying standard deviations ranging from 0.14 to 1.09, representing relatively large estimation uncertainty (for a more intuitive illustration, see boxplots of the estimates of all converged replicates on OSF: https://osf.io/698te/).

In conclusion, in the presence of 90% missing values, most conditions exhibited convergence issues. For converged replicates, the L-APIM handled the estimation of the fixed within-person effects well when there were a large number of measurement occasions per dyad (i.e., *K* = 70). However, estimating the between-person effects is more problematic, probably because the person averages are based on a few measurements only. In conditions with a small number of planned measurements per dyad (i.e., 14), relatively larger biases and SE values were observed. Consequently, we explored the performance of alternative models in the following section.

## 2 Examining alternative models

### 2.1 Overview of alternative models

We inspected the performance of alternative models that may simplify estimation and thus improve convergence, while having the potential to yield reliable estimates. The alternative models were obtained by simplifying the variance–covariance structures of residuals and random effects based on Gistelinck and Loeys (2019), and by investigating two frequently used modeling strategies. Table 5 summarizes the characteristics of all alternative models.

**Table 5:** Summary of alternative models

Random effects		Residuals			
			2. Heterogeneous	3. Homogeneous	4. Homogeneous
			variances with	variances with	variances with
		1. Unstructured	no correlation	correlation	no correlation
	A. Unstructured	1A: standard L-APIM 1A.S: estimate separately 1A.P: no partner effect	2A	3A	4A
	B. Heterogeneous variances with no correlation	1B	2B	3B	4B
	C. Homogeneous variances with correlation	/	2C	/	4C
Random intercepts and slopes	D. Homogeneous variances with no correlation	/	2D	/	4D
			1a			
		a. Unstructured	1a.S: estimate separately 1a.P: no partner effect	2a	3a	4a
		b. Heterogeneous variances with no correlation	1b	2b	3b	4b
	c. Homogeneous variances with correlation	1c	2c	3c	4c
Random intercepts only	d. Homogeneous variances with no correlation	1d	2d	3d	4d

*Note:* The combination of numbers and letters indicates different alternative models. The “/” symbol means that the combination cannot be estimated in R due to different clustering groups in residuals and random effects. For example, for type C, the clustering group is the individual, whereas for type 1 residuals, to capture correlation, the clustering group is the dyad.

#### 2.1.1 Simplifying variance–covariance structures of residuals and random effects

##### 2.1.1.1 Residuals

We restricted the variance–covariance structure of the residuals by assuming either equal variances (i.e., indistinguishability) and/or no correlation (i.e., non-interdependence) between partners’ residuals, yielding four possible structures.

1. Unstructured residuals, which allows for different residual variances for females ( 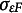 ) and males ( 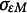 ), as well as a nonzero correlation ( 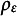 ), as specified in equation (4).
2. Heterogeneous residual variances combined with fixing the correlation of residuals between female and male partners to zero:
(5)

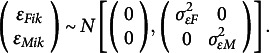

3. Homogeneous residual variances, which assumes equal residual variances for female and male partners, while still allowing for correlation between the residuals:
(6)

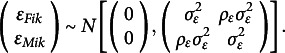

4. Homogeneous residual variances and uncorrelated residuals:
(7)

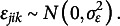

##### 2.1.1.2 Random effects

We also investigated eight types of variance–covariance structures for the random effects. Whereas the first four models include random parts for both intercepts and actor and partner effects, the last four only include random parts for the intercepts, which is a common analysis strategy (e.g., Gistelinck & Loeys, 2019; Savord et al., 2023). Both sets of four variance–covariance structures, A–D and a–d, closely resemble the four structures introduced for the residuals:

(A) Unstructured random intercepts and actor and partner effects, which allows for different variances for females and males as well as allowing their random effects to be correlated ( 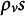 ), as specified in equation (3).

(B) Heterogeneous random intercept, actor and partner variances but uncorrelated random effects:
(8)

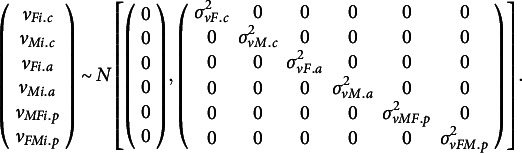



(C) Homogeneous random intercept, actor and partner variances with correlated random effects. To achieve this, we treated each individual as independent, while imposing their random effects to follow a multivariate normal distribution:
(9)

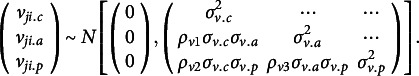



(D) Homogeneous intercept, actor and partner variances with no correlation of random effects, by further constraining equation (9):
(10)

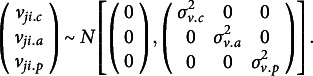

- Unstructured random intercepts:
(11)

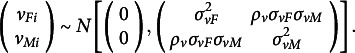

- Heterogeneous random intercept variances but imposing the random intercepts of males and females to be uncorrelated:
(12)

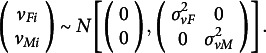

- Homogeneous random intercept variances with random intercepts being correlated:
(13)

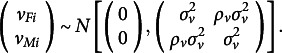

- Homogeneous random intercept variances and uncorrelated random intercepts:
(14)

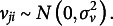

##### 2.1.1.3 Combining the different types of residual and random effect structures

A total of 32 models is obtained by fully crossing the four types of residuals and eight types of random effects. Since one of them boils down to the standard L-APIM, which utilizes type 1 residuals and type A random effects and is therefore labeled 1A, 31 of them are alternative approaches. However, due to practical reasons (i.e., different clustering structures for random effects and residuals), models 1C, 1D, 3C, and 3D cannot be estimated using “nlme”^3^. Therefore, only the other 27 models were investigated.

#### 2.1.2 Additional modeling strategies

In addition to these 27 models, we proposed four more alternative models. Specifically, we obtained two alternatives by fitting models 1A and 1a to the outcome scores of each partner separately (e.g., Planalp et al., 2017); we therefore label these approaches 1A.S and 1a.S. These alternatives indeed preserve the potential unstructured variance–covariance structures of residuals and random effects without estimating them, thereby simplifying estimation.

Next, we adopted a commonly used actor-only pattern by fixing all partner effects to zero while keeping the remaining random effects (i.e., intercepts and possibly slopes for within-person actor effects) and residuals unstructured (e.g., Hsueh et al., 2018; Kenny & Ledermann, 2010), yielding alternative models 1A.P and 1a.P. Notably, according to Kenny et al. (2006), an actor effect is defined as an effect estimated alongside a corresponding and simultaneous partner effect. Therefore, the term “actor effect” can be considered inappropriate when describing the actor-only models.

#### 2.1.3 Estimation

Consistent with the standard L-APIM estimation, we used pairwise deletion to handle missing values. We again opted for the “optim” optimizer and REML in the “lme()” function of the “nlme” package to estimate model parameters. The code to estimate all alternative models is available at https://osf.io/698te/.

### 2.2 Simulation results for alternative models

*Number of measurements included in estimation*. Actor-only models (1A.P and 1a.P), which remove all partner effects, led to an increase in the final sample size by including reports for which the report of the partner was missing. For the other models, results remained consistent with those for the standard L-APIM (see Table 4).

**Figure 4:**
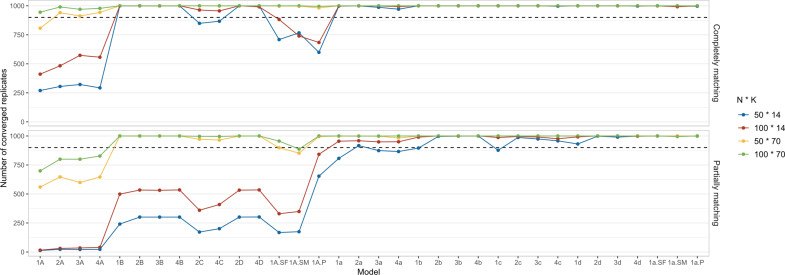
Number of converged replicates across 1,000 replicates. *Note*: The dashed line indicates 90% acceptable convergence rate. Models from “1A” to “1A.P” are multilevel models with random intercepts and slopes. Models from “1a” to “1a.P” are random-intercept-only models. “1A.SF” and “1A.SM” represent separate estimation for females and males, respectively.

*Convergence*. Figure 4 shows the number of converged replicates for each alternative model. Overall, most models significantly improved convergence. We found that manipulating the variance–covariance matrix of residuals (i.e., choosing from type 1 to 4) led to limited improvement, whereas altering the variance–covariance matrix of random effects showed a larger impact. Specifically, for models with random intercepts and random actor and partner effects, type B (heterogeneous variances with no correlation) and type D (homogeneous variances with no correlation) performed the best and showed acceptable convergence rates in most conditions, except when the sample size was very small (*K* = 14 and partially matching). Type C (homogenous variance with correlation) performed less well but still improved convergence, while type A (unstructured) typically did not improve convergence substantially. When including only random intercepts (i.e., type a to d), almost all combinations of random effects and residuals led to acceptable convergence rates, except for the combination of a few more complex models (e.g., 1a, 3a…) with small sample size (50 * 14 and partially matching).

Estimating separately for females and males (1A.S) can improve convergence more than using other type A models but less than types B to D models. Removing the partner effect (1A.P) can significantly improve convergence in partially matching conditions, outperforming other alternative models with random intercepts and random actor and partner effects. However, this superiority is less pronounced in completely matching conditions, which makes sense as sample size is then unaffected by dropping the partner effects. The random-intercept-only extensions for both modeling strategies almost always converged.

**Figure 5:**
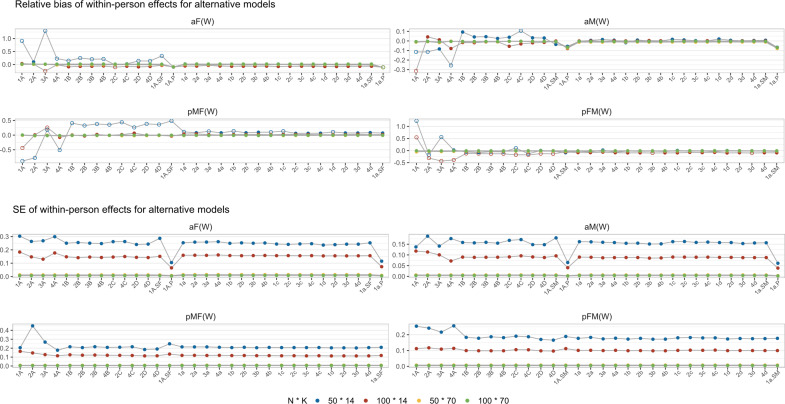
Relative bias and SE for alternative models. *Note*: The open dots on the upper four grids represent biases larger than 10%. We plotted the results from actor-only models (1A.P and 1a.P) in the actor effect panels for simplicity (also in Figures 6 and 7), but it is important to note that these results no longer represent actor effects.

**Figure 6:**
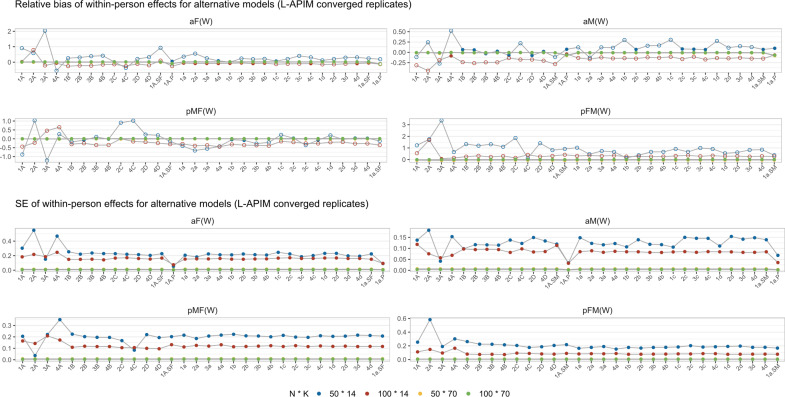
Relative bias and SE for alternative models (L-APIM converged replicates).

*Relative bias and SE*. Generally, we observed minimal variation in the estimation across different models within each replicate. The results for all converged replicates are displayed in Figure 5, whereas Figure 6 focuses only on the replicates that converged for the standard L-APIM model. To simplify the interpretations, we focused on the fixed within-person effects, as they typically are the main focus of ILD studies, and on partially matching conditions due to more serious missing data and non-convergence issues. The results for between-person effects and completely matching conditions can be found in the Supplementary Material, and the boxplots of the estimation of all converged replicates can be found on OSF: https://osf.io/698te/. The biases in between-person effects were not improved by alternative models.

Regarding relative bias, no significant biases were observed when *K* = 70. However, when *K* = 14, some biases larger than 10% were observed, particularly in models with both random intercepts and random actor and partner effects. In these conditions, the use of random intercepts only models but with partner effects seems to be recommended. The removal of the partner effects leads to consistent slight biases.

Comparing the SEs across all alternative models, we found that the SE values were larger with smaller sample sizes. Most alternative models provide similar performance, except for some fluctuations within models with type A random effects, which might be due to their larger non-convergence issues. Additionally, the 1A.P and 1a.P models (removing partner effects) exhibited the lowest SE values. This result is due to the larger sample size in partially matching conditions. The superiority of 1A.P and 1a.P was no longer observed for completely matching conditions (as shown in the Supplementary Material).

### 2.3 Fitting alternative models to the daily diary data

We fitted the alternative models to the 18 combinations of predictors and outcomes in the diary data, and examined whether the simulation findings can be generalized to real-world data. We evaluated the performance of the alternative models in two aspects. First, we explored whether the observed improvements in convergence for alternative models aligned with those found in simulations. Second, we aimed to compare the obtained parameter estimates, acknowledging that the true effect values remain unknown. The example dataset consisted of 75 dyads with 14 days. Pairwise deletion of days where at least one of the partner reports was missing, resulted in a total data loss of 90.76%. We consider this level of data loss comparable to the completely matching conditions in the simulations. The simulation study revealed that even though we encountered convergence issues in completely matching conditions with sample sizes of 50 × 14 and 100 × 14, the L-APIM estimates in converged replicates showed no significant bias for within-person effects and exhibited relatively small SE values. Consequently, we deemed the standard L-APIM estimates to provide a good benchmark for the comparison with the alternative models.

**Figure 7:**
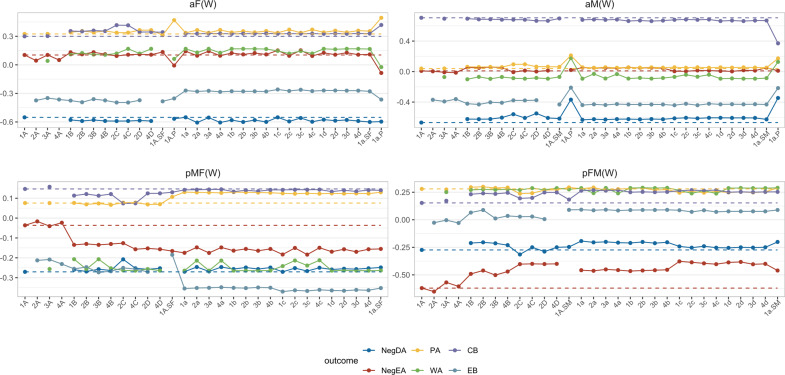
Estimates obtained from alternative models in daily diary data (predictor = Autonomy). *Note*: NegDA, Negative Disengaging emotion; NegEA, Negative Engaging emotion; PA, Positive emotion; WA, Worry; CB, Considerate behavior; EB, Evasive behavior. The dashed lines show the estimates from the standard L-APIM.

Figure 7 shows the estimated within-person effects for models with autonomy as the predictor, the other results can be found in the Supplementary Material. Overall, we found that, except for models with type A random effects, all alternative models showed better convergence rates. This aligned with our simulation findings that these models have the potential to substantially improve convergence. Regarding estimates, type A models deviated the least from the standard L-APIM (model 1A), but results were otherwise generally stable across all alternative models that included partner effects.

## 3 Discussion

The aim of this study was twofold. First, we aimed to explore the performance of the L-APIM and the potential statistical challenges encountered when applied to dyadic ILD with large amounts of missing values. Second, we wanted to evaluate whether alternative models could address the statistical challenges of the L-APIM and be feasible options.

Convergence issues were observed when applying the L-APIM to the daily diary dataset. In addition, using a simulation design, we found that L-APIM underperformed under certain circumstances. More specifically, the average convergence rates varied from 1.3% to 94.50%. Higher convergence rates were found to be associated with higher levels of matching and larger sample size. Regarding relative biases and SE of estimation, the estimation of fixed within-person effects was generally unbiased, except for partially matching conditions with fewer measurement occasions (*K* = 14). However, we found relative biases larger than 10% across most conditions for the between-person effects, which is consistent with previous findings that the calculated time-average component may not reliably reflect the unobserved person average in case the sample size per person is small (e.g., Lüdtke et al., 2008; Raudenbush et al., 1991). Smaller SE values were observed with larger sample size and in completely matching conditions, whereas relatively large SE values were found when *K* = 14, indicating uncertain estimation.

Though seemingly extreme, the amounts of missing data studied are not unrealistic. For instance, in the study by Derrick and Testa (2017), male verbal aggression was reported only 34 times by both partners among 157,540 reports provided by couples, indicating 0.02% of valid data. Similarly, in the daily diary dataset used in the present study, 90.76% of the data was lost due to mismatching reports between partners. In summary, when the data contain many missing values, the use of L-APIM might lead to non-convergence, implying that alternative models should be considered. However, as long as there are sufficient measurements per person, whenever the L-APIM converges, the L-APIM remains recommended for estimation of the within-person effects, even if data loss is high.

To handle non-convergence issues, 31 alternative models were explored. On the statistical side, most models indeed demonstrated improved convergence, except for those with unstructured random effects (type A). Across the different variance–covariance structures of residuals and random effects, and for the approaches that estimated the model for both partners separately (1A.S and 1a.s), simulations did not reveal clear biases or larger SE for within-person effects. However, we also did not find a clear improvement in the estimation of between-person effects, or a reduction in the uncertainty of within-person effect estimation when the sample size is small (*K* = 14). Models that do not include partner effects (i.e., actor-only models) provide low SE of within-person effects in partially matching conditions, solely due to the increased sample size. However, we emphasize that this comes at the cost of inherent bias in the estimation of within-person effects. This is also found in the empirical analyses of the daily diary data, where the actor-only model estimates deviated substantially from those of other models. Therefore, unless the strong assumption of no partner effects can be expected to hold, we consider the “actor-only” model a suboptimal and misspecified alternative. Empirical analyses of the other models for the daily diary data revealed a similar pattern, but also showed some slight differences between the estimates of type A models and other models.

Conceptually, except for models 1A.S and 1a.S, which separately estimate effects for females and males but are conceptually equivalent to the standard L-APIM, all other models impose additional constrains on certain parameters. These constraints assume independence of partners’ residuals or random effects, indistinguishability of residual or random effect variances, or non-existence of partner effects, which can lead to estimation biases. Exploring whether the effects of these constraints on estimates for empirical data are consistent across different models can shed light on which effects are likely present in the data.

In addition to addressing missing data issues by fitting multiple alternative models, it is worthwhile to prevent these issues beforehand. Given the impact of a larger valid sample size on reducing estimation uncertainty, improving convergence and statistical power (as detailed in the Supplementary Material), it is crucial to obtain an adequate sample size with higher compliance and more matching measurements through optimized study designs.

In summary, our findings support the idea that large amounts of missing data may impede the effectiveness of L-APIM estimation and highlight the necessity for sufficient sample size. While the L-APIM is generally recommended for estimating within-person actor and partner effects in common real-life data, alternative models can be considered when the L-APIM fails to converge. Overall, we suggest fitting multiple alternative models to examine the robustness of conclusions.

### 3.1 Limitations

This study has some limitations. First, we considered the L-APIM as the “right” model and chose the alternative models based on them being simplified versions of the L-APIM. Other models have been proposed for dyadic ILD (Iida et al., 2023), which focus on different research questions. Examples are the dyadic growth curve model (DGCM; e.g., Raudenbush et al., 1995) and the common fate growth model (CFGM; e.g., Kenny & La Voie, 1985; Ledermann & Macho, 2014), while it remains unclear to what extent these models are robust to missing values in dyadic ILD. Another limitation regarding model selection concerns the alternative models. We acknowledge that we did not exhaustively explore all potential alternative models. Therefore, there could be feasible alternative models not explored in this article that warrant further investigation.

Second, there is some literature on how to test the viability of some of the constraints that are imposed by the alternative models. For example, Kenny et al. (2006) demonstrated methods to assess interdependence and distinguishability between partners, and the test of *k* ratio (the partner effect divided by the actor effect) proposed by Kenny and Ledermann (2010) can provide insight into whether the partner effects are minimal and whether using the actor-only pattern in empirical estimation is appropriate. We refrained from such tests in this article as they are of less use when the standard L-APIM model cannot be estimated and hence no benchmark model is available.

Third, although we aimed to simulate data that are comparable to real datasets, there are still real-world characteristics that we did not fully capture. For example, in our simulation, we removed 90% of data, assuming it did not reflect specific contexts. Moreover, in the partially matching condition, we allowed for mismatches between partners, which led to additional missingness occurring completely at random (MCAR). However, in real-life scenarios, the occurrence of disagreements and the congruence in partners’ reports about them may be influenced by various factors. For instance, partners may only agree on more intense disagreements. Future studies could explore different missing data mechanisms in greater depth and examine their consequences for estimation.

Fourth, this article was motivated by the statistical issues we encountered in the daily diary data. Yet the parameters derived from the data could be different from those in other datasets, potentially influencing the results. To assess the generalizability of our findings, we examined performance of L-APIM and alternative models and conducted simulations with parameters from an additional ESM dataset (see details in Supplementary Material). The conclusions remained consistent, further supporting our findings that statistical issues could be generally relevant in the presence of large amounts of missing data and affirming the feasibility of alternative models.

Fifth, relatively larger biases were observed when estimating the between-person effects. These biases could not be handled by using alternative models. Future research could explore the underlying mechanisms of such biases and develop strategies to mitigate them.

Sixth, we opted for pairwise deletion to handle missing values, as it is straightforward and commonly used, especially when the missing percentage is substantial and imputation methods are deemed inappropriate (Jakobsen et al., 2017). However, such deletion method may result in significant data loss, especially when there are few matching reports, thereby reducing statistical power. Additionally, pairwise deletion assumes missing completely at random (MCAR) and might introduce biases if this assumption does not hold in real-world data. Future research could explore alternative strategies for handling missing data, such as full information maximum likelihood (FIML) in structural equation modeling (SEM), which utilizes all available data for estimating each effect with the less restrictive assumption of missing at random (MAR).

## 4 Conclusion

Overall, the present study explored the statistical challenges of using L-APIM models for analyzing dyadic ILD with large amounts of missing values, and provided insight into how to handle the challenges by using alternative models. These findings can contribute to the methodological understanding and practical application of longitudinal dyadic analyses and highlight the importance of sufficient sample size.

## Supporting information

Ji et al. supplementary material Ji et al. supplementary material

## Data Availability

The daily diary data analyzed during the current study are available in the Open Science Framework repository at https://osf.io/s4qm3/. The dataset generated during the study and all the code files are available in the Open Science Framework repository at https://osf.io/698te/.

## Footnotes

1
Other data structures can be used to estimate L-APIM using methods such as structural equation modeling (SEM) and Mplus. These include the commonly used “wide format,” where each dyad’s data is contained in a single row with separate columns for each partner’s variables, and the “long unstacked format,” which does not require the extra column storing the predictor from the partner (Walther et al., 2024).

2
The results of statistical power (for the L-APIM and alternative models) can be found in the Supplementary Material.

3
These models can be estimated using other software (e.g., Mplus) or modeling strategies (e.g., Bayesian estimation).

## Supplementary material

To view supplementary material for this article, please visit http://doi.org/10.1017/psy.2025.18.
