# Supplementary material for: Performance of the Longitudinal Actor–Partner Interdependence Model in Case of Large Amounts of Missing Values: Challenges and Possible Alternatives
Source: Psychometrika. 2025 Jun 13;90(2):508–30. doi: 10.1017/psy.2025.18 (PMC12483710; doi:10.1017/psy.2025.18)

Supplementary material

Table S1

Variable details of daily dairy dataset

Relationship goal (achievement)	Emotion	Behavior
1. Relatedness	***1. Negative disengaging***	***1. Considerate behavior***
felt close to each other	Sad	I listened to my partner’s point of view
were loyal and committed to each other	Hurt	I showed understanding towards my partner
maintained harmony in the relationship	Annoyed	I guessed what my partner was feeling
felt that we cared about each other	Disappointed	I acted annoyed towards my partner
felt connected to each other	Abandoned	***2. Evasive behavior***
2. Social roles	Rejected	I withheld potentially upsetting information from my partner
behaved as others around us would expect from a husband/wife/partner	***2. Negative engaging***	I discussed with my partner openly
fulfilled our roles as a husband/wife/partner	Ashamed	I did nothing and left the matter to chance
3. Autonomy	Guilty	I ignored my partner
stood up for our own needs	Embarrassed	I hid or denied my negative feelings
could both pursue our goals	***3. Positive emotion***	I did not involve myself with my partner until the tension settled down
expressed who each of us really was	Calm	***Removed***
could act and think freely	Strong	I acted more positive toward my partner than I felt
could make our own decisions as a couple	Proud	I refused to reconsider my own opinion
Removed	Secure	I sensed the atmosphere between us
conformed to each other’s wishes	***4. Worry***	I told my partner what he/she did wrong
could be the couple we wanted to be	Worried	I gave up on the argument
changed our decisions according to each other’s wishes	Concerned	
	Removed	
	Like my partner would indulge any of my requests	
	Empathy	
	Resigned	

Note. The scales were based on principal component analyses, some items were removed due to component loadings smaller than 0.40.

Comparison of estimation methods

We conducted a preliminary simulation study to compare different estimation methods (i.e., Bayesian estimation using the “brms” package, and the “optim” and “nlminb” optimizers in the “nlme” package).

Data generation

The data was simulated following two steps.

Step 1: generate complete data. We generated a complete dataset following the random-intercept-only model without separating between- and within-person effects. In this model, the outcome scores of each dyadic partner (Y_{Fik} and Y_{Mik}) are predicted based on their own predictor scores (resp. X_{Fik} or X_{Mik}), yielding the actor effects a_F and a_M, and those of their partner, implying the partner effects p_M and p_F:

$$Y_{Fik} = c_F + \nu_{Fi} + a_F X_{Fik} + p_{MF} X_{Mik} + \epsilon_{Fik} \tag{S1}$$

$$Y_{Mik} = c_M + \nu_{Mi} + a_M X_{Mik} + p_{FM} X_{Fik} + \epsilon_{Mik} \tag{S2}$$

Both the fixed (c_F and c_M) and random (ν_{Fi} and ν_{Mi}) intercept parts of the two partners are allowed to differ. The random intercepts are assumed to be bivariate normally distributed:

$$\begin{pmatrix} \nu_{Fi} \\ \nu_{Mi} \end{pmatrix} \sim N\left[\begin{pmatrix} 0 \\ 0 \end{pmatrix}, \begin{pmatrix} \sigma_{\nu F}^2 & \rho_\nu \sigma_{\nu F} \sigma_{\nu M} \\ \rho_\nu \sigma_{\nu F} \sigma_{\nu M} & \sigma_{\nu M}^2 \end{pmatrix}\right] \tag{S3}$$

Additionally, the Level 1 errors (ϵ_{Fik} and ϵ_{Mik}) are assumed to be bivariate normally distributed:

$$\begin{pmatrix} \epsilon_{Fik} \\ \epsilon_{Mik} \end{pmatrix} \sim N\left[\begin{pmatrix} 0 \\ 0 \end{pmatrix}, \begin{pmatrix} \sigma_{\epsilon F}^2 & \rho_\epsilon \sigma_{\epsilon F} \sigma_{\epsilon M} \\ \rho_\epsilon \sigma_{\epsilon F} \sigma_{\epsilon M} & \sigma_{\epsilon M}^2 \end{pmatrix}\right] \tag{S4}$$

The parameters used for data generation was derived by fitting the model to the daily diary data. Here we manipulated three factors, including the number of dyads, the number of measurement occasions per dyad, and the values of the actor and partner effects (see Table S2). Adapting the data simulation function from the paper by Lafit et al. (2021), we sampled the predictor scores of both partners as well as their level 1 errors and random intercepts from bivariate normal distributions as specified in Table S2. Using the L-APIM equations (S1) and (S2), these predictor scores and level 1 errors were combined into the outcome scores.

Step 2: introduce missing values. To mimic the data loss, we removed 90% of the data following different procedures. Specifically, we manipulated a fourth factor: matching pattern. The random condition implies removing measurement days for each partner separately (i.e., partner reports on disagreement are independent), while the matching condition indicates that exactly the same measurement days are missing for each partner (i.e., they perfectly agreed about the occurrence of a disagreement).

Table S2

Manipulated factors and for comparing estimation methods

Parameter	Value for data generation
Number of dyads (N)	[50; 100]
Number of measurements per dyad (K)	[14; 70]
Matching pattern	[matching; random]
Fixed effect (a_F, a_M, p_{MF}, p_{FM})	[(0.5, 0.5, 0.3, 0.3);<br>(0.5, 0, 0.3, 0);<br>(0.5, 0.5, 0, 0);<br>(0.3, 0.3, 0.1, 0.1);<br>(0.3, 0, 0.1, 0);<br>(0.3, 0.3, 0, 0);<br>(0, 0, 0, 0)]
Fixed effect (c_F, c_M)	(1.40, 0.65)
Standard deviation of the residual for female and male ($\sigma_{\epsilon F}$, $\sigma_{\epsilon M}$)	(1.00, 1.00)
Correlation between the residual of female and male(ρ_ϵ)	0.10
Standard deviation of the random intercepts for female and male (σ_{vF}, σ_{vM})	(0.60, 0.60)
Correlation between the random intercepts of female and male (ρ_v)	0.6
Mean and standard deviation of the predictor of female and male (X_F, X_M, SD_F, SD_M)	(4, 4, 1, 1)
Correlation between the predictor of partner female and male (ρ_X)	0.1

Note. Brackets for the first four rows indicate conditions for the manipulated factors.

Estimation

Random-intercept-only L-APIMs were fitted using different estimation methods to simulated datasets across 56 conditions, each with 100 replicates. The 56 conditions resulted from fully crossing the four factors.

Results

Convergence. The number of converged replicates of the three estimation methods is shown in Figure S1. Notably, we identified the convergence of Bayesian estimation by evaluating the Rhat values (Gelman & Rubin, 1992). Rhat measures the ratio of the average variance within each chain to the variance across all chains. If the chains haven't converged to a common distribution, Rhat will be greater than one. We considered Rhat values exceeding the thresholds of 1.1 and the more conservative 1.05 as indicative of non-convergence (both thresholds are provided for reference). We found that the “optim” optimizer in the “lme()” function showed good convergence, comparable to Bayesian estimation when using the easier 1.1 threshold.

Estimation. We then evaluated the estimation of fixed effects. As an illustration, Figure S2 presents the estimation of the actor effect for female partners under the condition of fixed effects (0.5, 0.5, 0.3, 0.3). Overall, we found that the estimation results of all methods did not differ significantly from each other.

Therefore, considering the superiority in convergence of the “optim” optimizer and its computational efficiency (usually less than 1 second) compared to Bayesian estimation (around one minute), we chose the "optim" optimizer for the paper.

Figure S1

Number of convergence

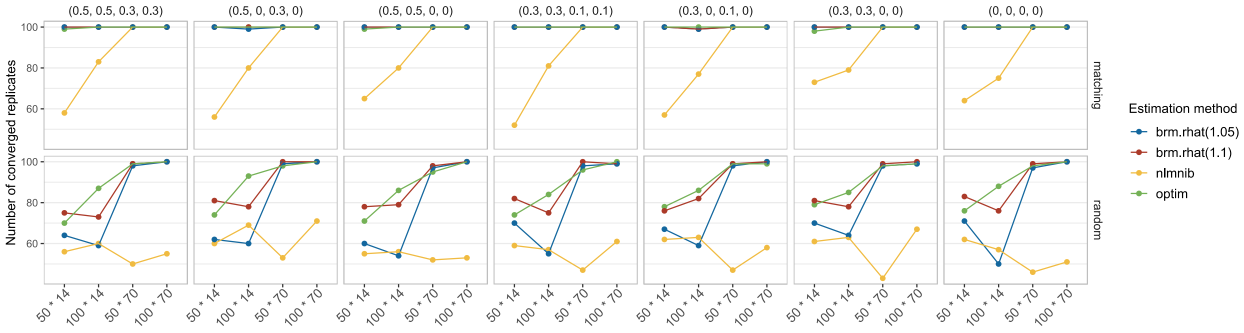

Note. Numbers in parentheses indicate the values of fixed effects (a_F, a_M, p_{MF}, p_{FM}).

Figure S2

Estimation of actor effect of female partners

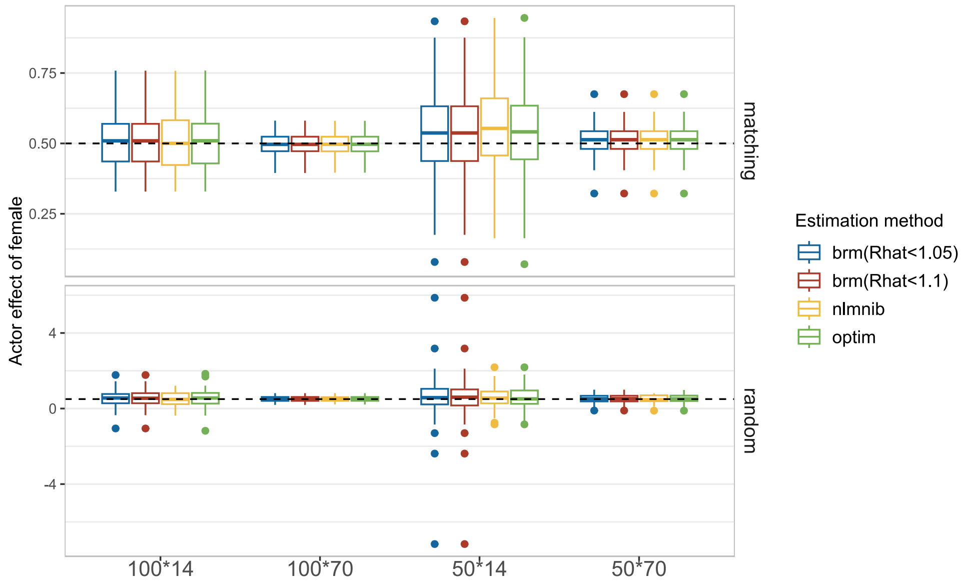

Note. The dashed lines indicate the true value (0.5) of actor effect of female partners

Statistical power

Statistical power was measured as the proportion of Monte Carlo replicates in which the actor/partner effect was significant (at $\alpha = 0.05$). The results are shown in Figure S3. Generally, higher statistical power was associated with larger sample sizes and higher true effects. In partially matching conditions, model 1A.P and 1a.P (removing partner effect) exhibited the highest power for estimating actor effect, which makes sense due to the inclusion of larger sample sizes. Among other alternative models, the statistical power of within-person effects remained relatively stable. In contrast, some differences were observed in between-person effects. Specifically, two distinct groups of models emerged: models of types A, B, a, and b demonstrated similar performance, while models of types C, D, c, and d showed relatively consistent performance within their group. These two groups typically differed from each other when estimating between-person effects.

Figure S3

Statistical power for alternative models

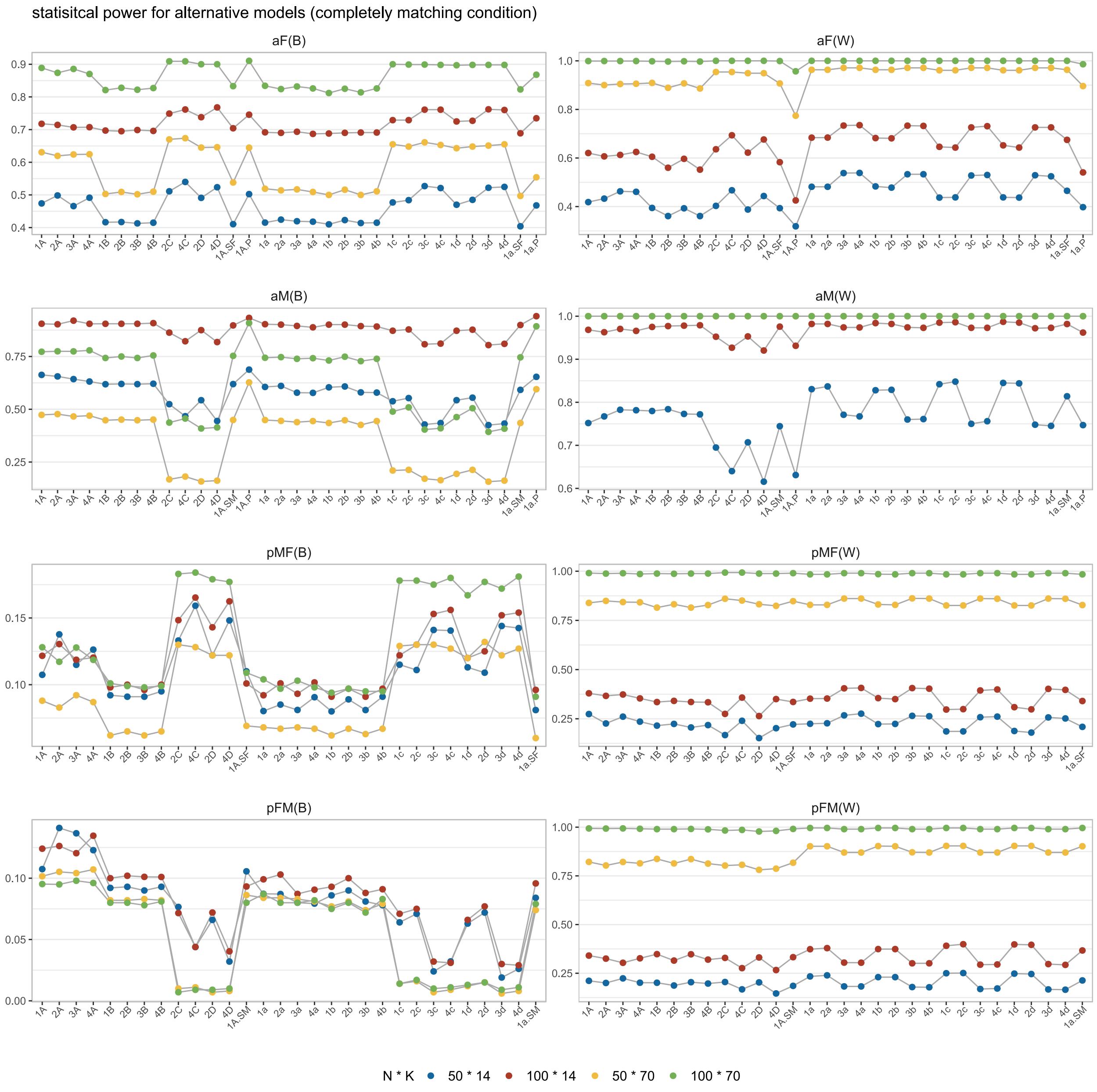

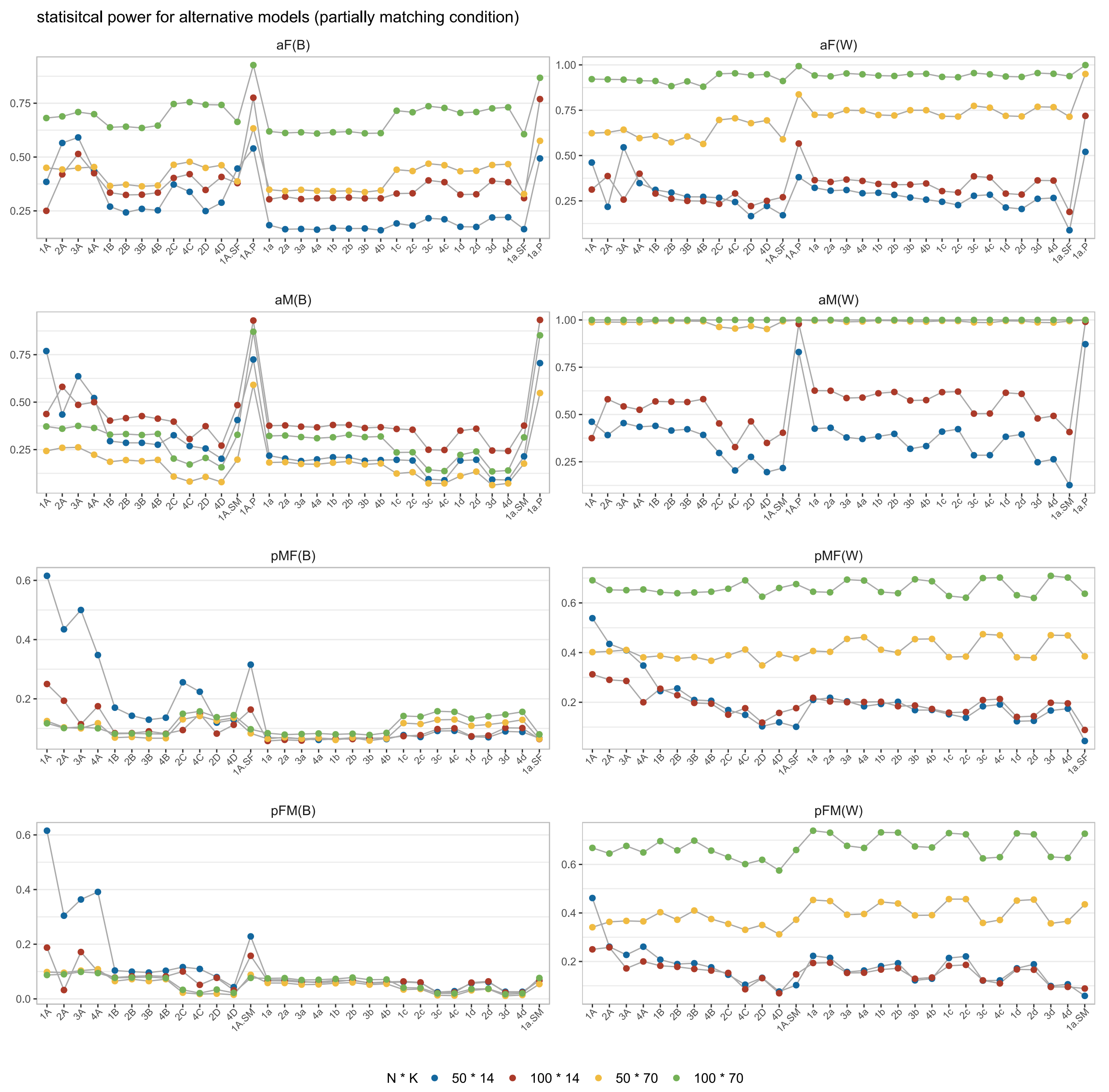

Note. “B” in the parentheses indicate between-person effects, while “W” indicates within-person effect. The true values of all effect are $a_{F(B)}$ = -0.6; $a_{F(W)}$ = -0.6; $a_{M(B)}$ = -0.1; $a_{M(W)}$ = -0.7; $p_{MF(B)}$ = 0.3; $p_{MF(W)}$ = -0.3; $p_{FM(B)}$ = -0.1; $p_{FM(W)}$ = -0.3.

Relative bias and SE for alternative models

In the paper, we presented the results of within-person effects in partially matching conditions. Here, we provide the remaining results.

Figure S4

Relative bias and SE for alternative models

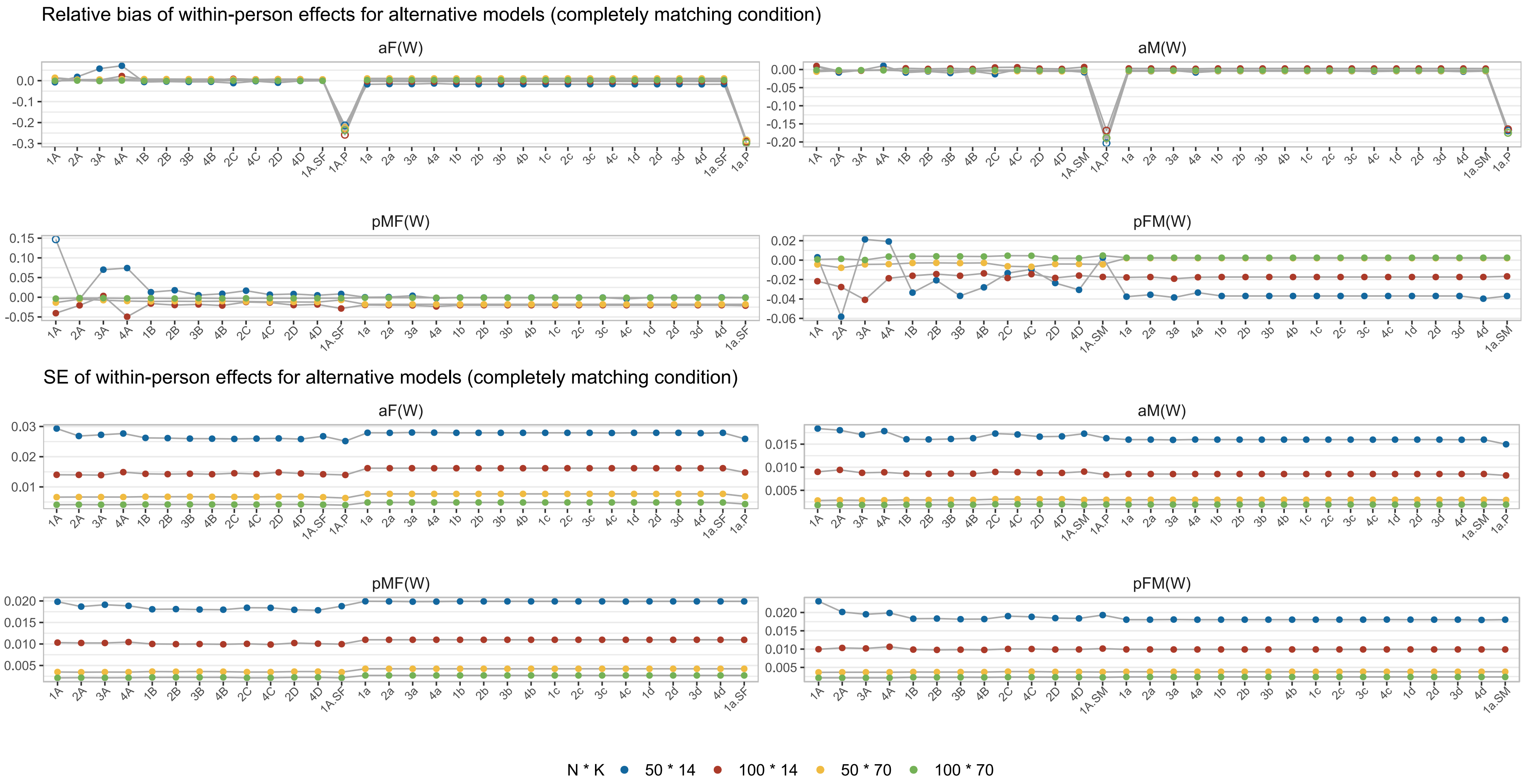

Relative bias of between-person effects for alternative models (completely matching condition)

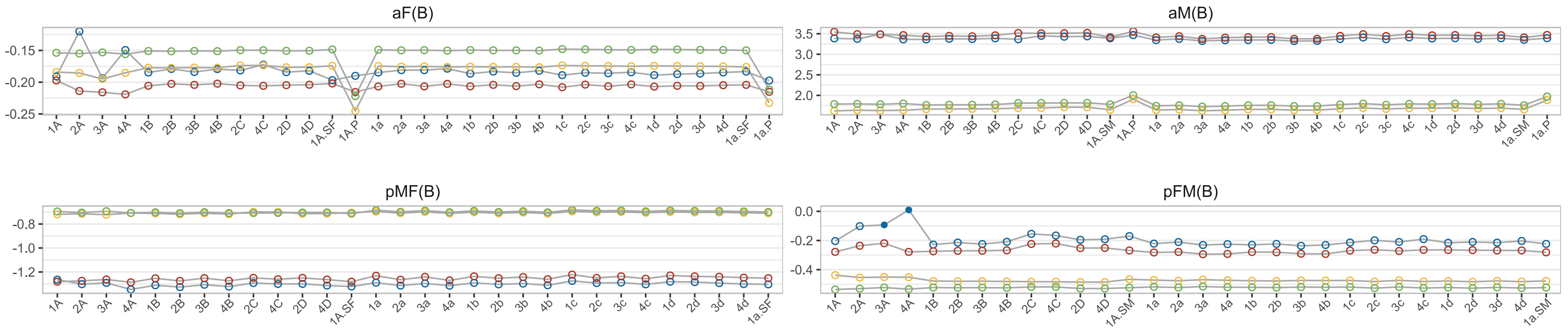

SE of between-person effects for alternative models (completely matching condition)

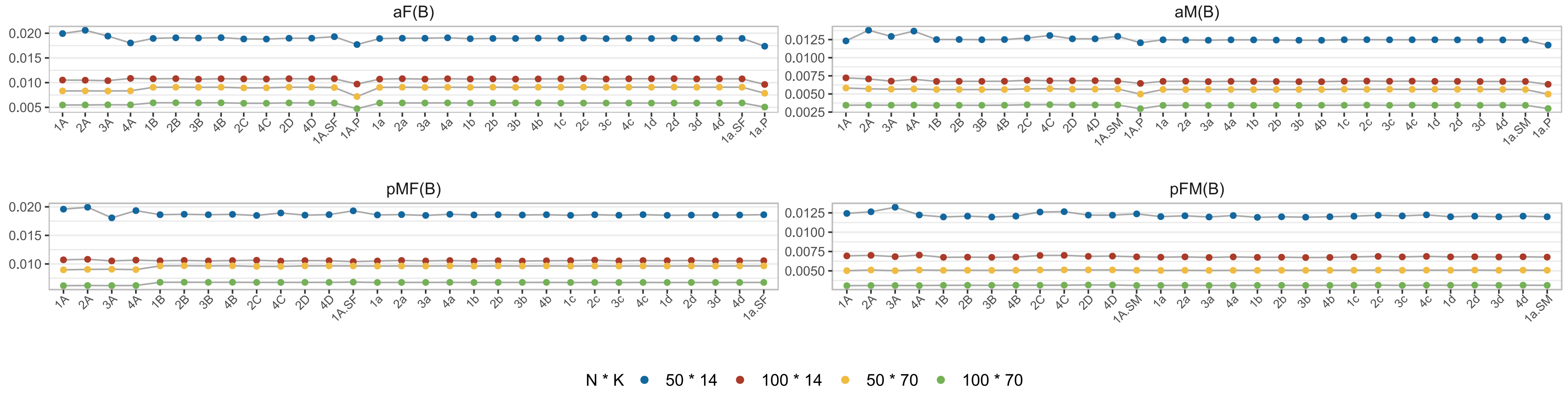

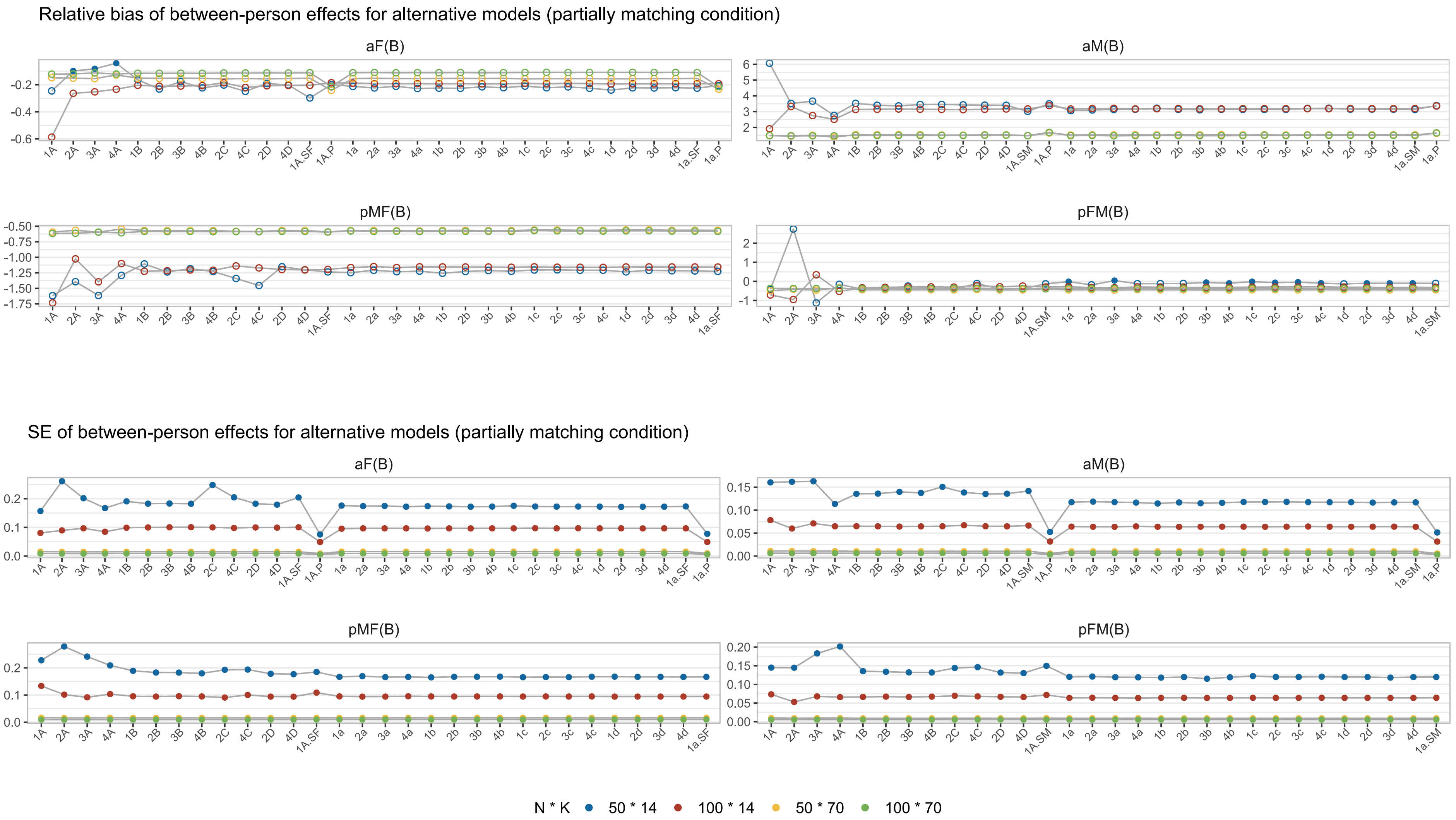

Note. The open dots on the upper four grids represent biases larger than 10%.

Fitting alternative models to daily diary data

In the paper, we presented the results of within-person effects when the predictor is autonomy. Here, we present the remaining results.

Figure S5

Estimates obtained with alternative models

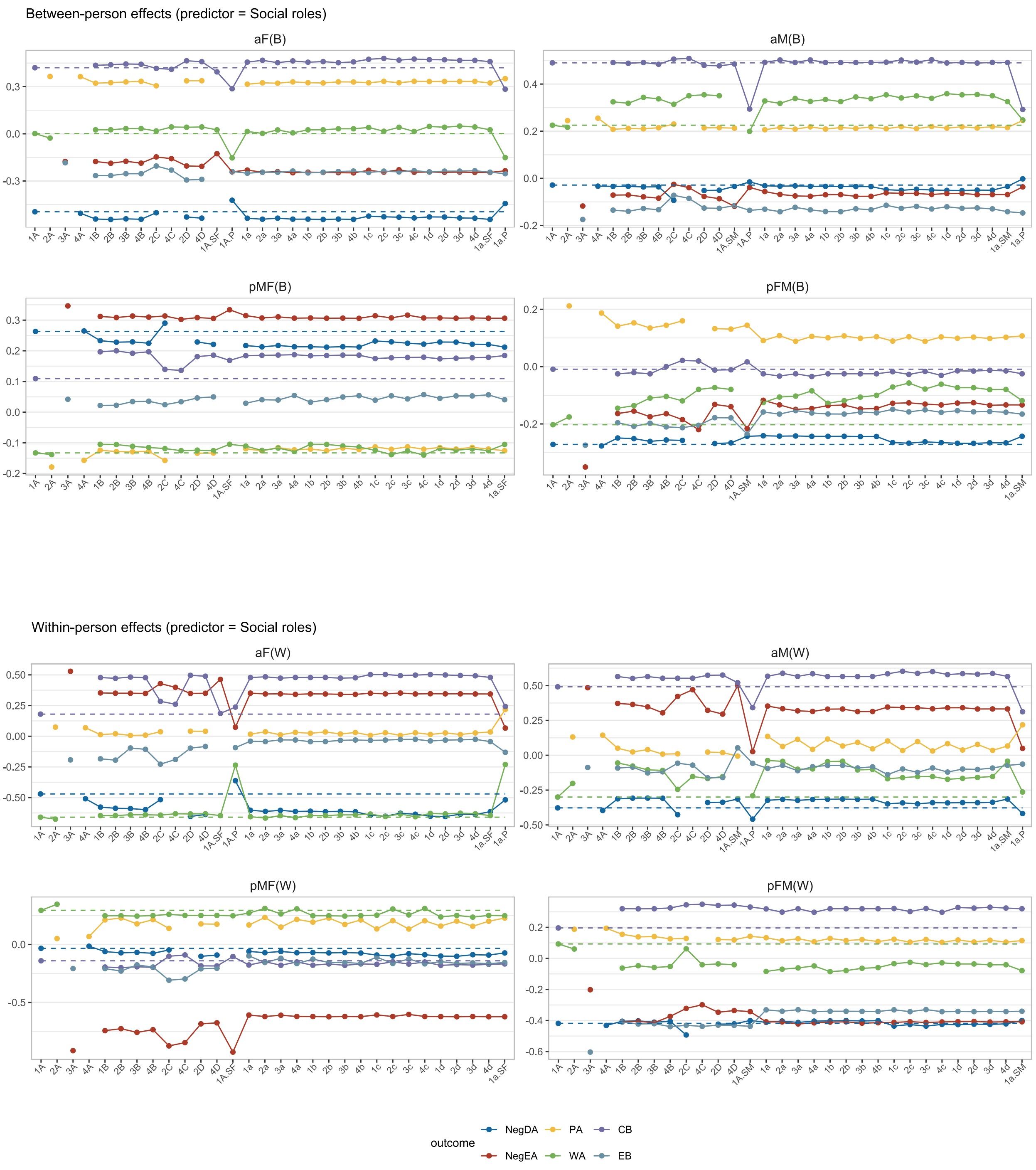

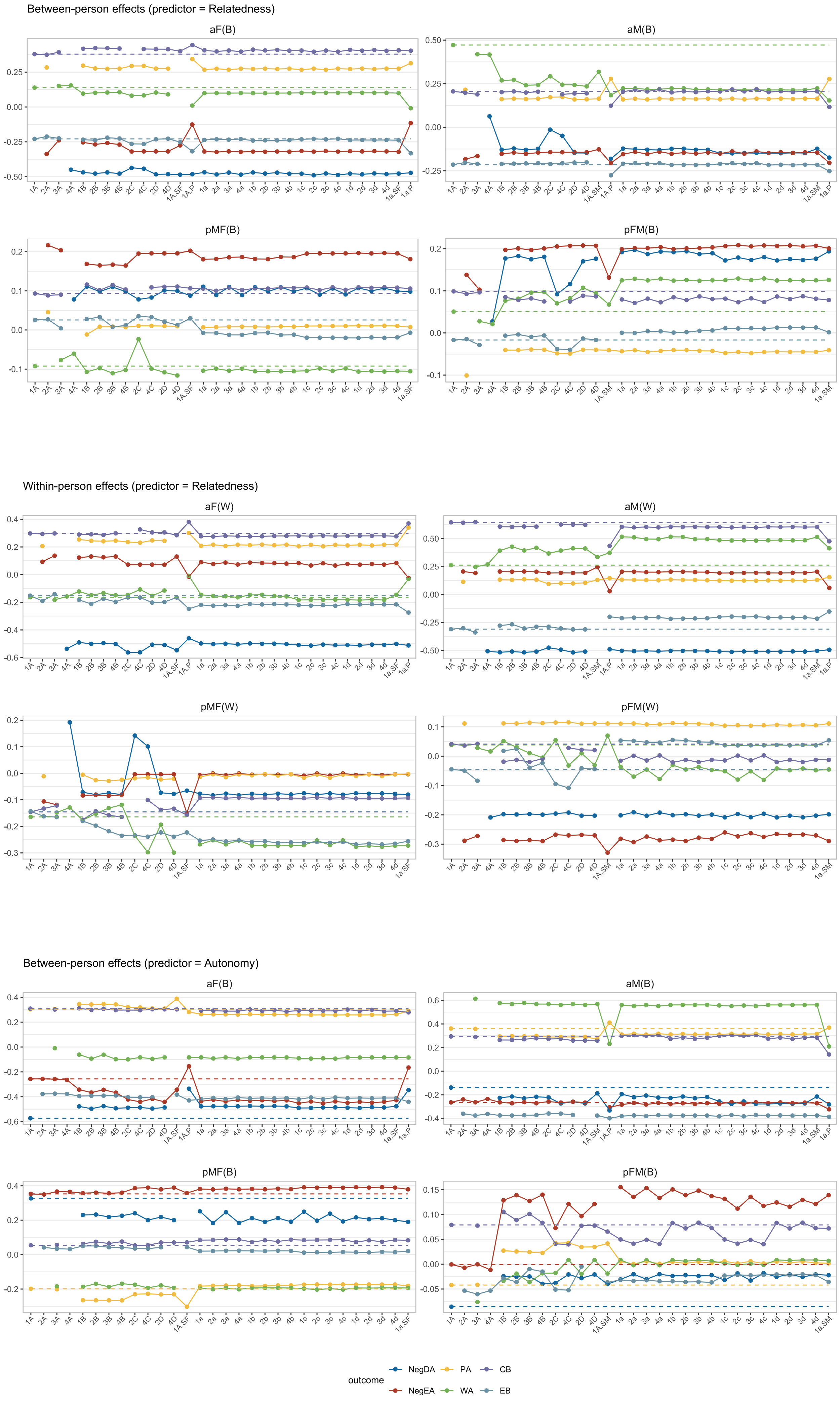

Note. NegDA = Negative Disengaging emotion, NegEA = Negative Engaging emotion, PA = Positive emotion, WA = Worry, CB = Considerate behavior, EB = Evasive behavior. The dashed lines show the estimates from the standard L-APIM.

Examining the generalizability of findings: ESM data

The considered ESM data was collected from 50 couples (M_{age} = 27.75, SD_{age} = 10.60), with 10 measurement occasions a day for 7 consecutive days. On each beep, participants were asked whether they and their partner talked about problems they encountered. Their emotions (anger, anxiety, depression, sadness, relaxation, satisfaction, happiness, and cheerfulness), as well as their stress and self-confidence were measured using slider scales ranging from 0 to 100. When only focusing on the beeps where partners discussed problems together, there was again a substantial reduction in sample size (13.30 measurements per participant), and even fewer matching reports (7.98 per couple). We fitted the data using both the L-APIM and alternative models. Parameters extracted from the dataset were used to conduct simulations. To simplify interpretation, we present the results in two sections: first, the empirical results of both the L-APIM and alternative models, and second, the simulation results.

Fitting L-APIM and alternative models to ESM data

We chose the eight emotions as outcomes and stress and self-confidence as predictors, yielding a total of 16 L-APIM models (model 1A as specified in the paper). Among these 16 models, 5 failed to converge (see Figure S6 for the results of female's actor effect), supporting that non-convergence could occur in real data, especially, when there are many missing values.

When comparing alternative models. Similarly, except for models with type A random effects, all alternative models improved convergence. Regarding estimates, type A models deviated the least from the standard L-APIM (model 1A), but results were otherwise generally stable across all alternative models that included partner effects.

Figure S6

Estimates obtained with L-APIM and alternative models (ESM data)

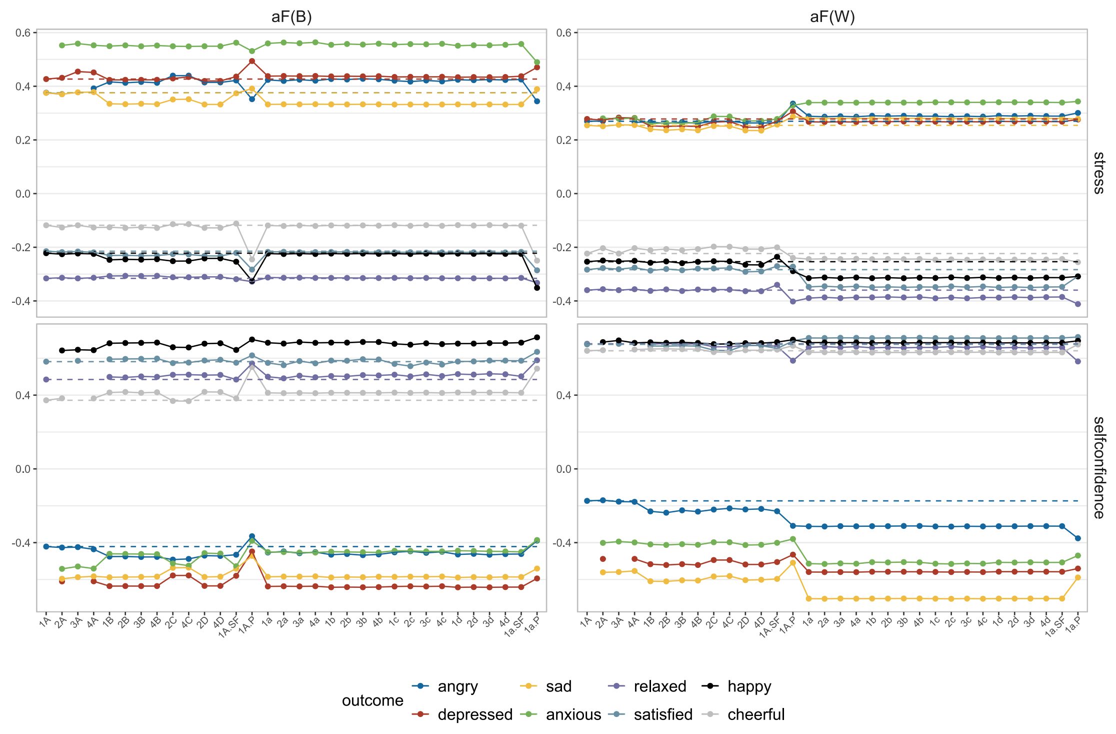

Simulation results

The data was simulated following the two steps outlined in the paper. The parameters used for data generation were derived from the ESM data and are detailed in Table S3. The same three factors (i.e., *N, K*, and matching pattern) were manipulated, yielding eight conditions, each with 1000 replicates.

Table S3

Parameters for data generation (ESM data)

Parameter		Value for data generation					
Number of dyads (N)		[50, 100] (50)					
Number of measurements per dyad (K)		[14, 70] (70)					
Missing percentage		90% (91.33%)					
Matching pattern		[completely matching, partially matching]					
Fixed effects		c_F	$a_{F(B)}$	$a_{F(W)}$	$p_{MF(B)}$	$p_{MF(W)}$	
		3.9	0.2	0.2	0	0.1	
		c_M	$a_{M(B)}$	$a_{M(W)}$	$p_{FM(B)}$	$p_{FM(W)}$	
		1.8	0.4	0.2	0	0	
Grand mean for predictor		c_{XF}	c_{XM}				
		22.8	20.1				
SD and correlation of		*SD*	correlation				
Residuals	ϵ_{Fit}	16.45	ϵ_{Fit}				
	ϵ_{Mit}	14.33	0.09				
Random effects	$\nu_{Fi.c}$	6.67	$\nu_{Fi.c}$	$\nu_{Mi.c}$	$\nu_{Fi.a}$	$\nu_{Mi.a}$	$\nu_{MFi.p}$
	$\nu_{Mi.c}$	5.76	0.36				
	$\nu_{Fi.a}$	0.13	0.83	0.57			
	$\nu_{Mi.a}$	0.19	0.14	0.62	0.40		
	$\nu_{MFi.p}$	0.09	0.58	0.15	0.44	0.26	
	$\nu_{FMi.p}$	0.08	0.42	-0.08	0.41	0.17	
Time-specific part for predictor	ϵ_{XFit}	22.1	ϵ_{XFit}				
	ϵ_{XMit}	19.7	0.18				
Time-average part for predictor	ν_{XFi}	14.3	ν_{XFi}				
	ν_{XMi}	12.6	0.4				

Note. Numbers in the brackets for the first three rows indicate the parameter values from the empirical data. The other parameter values used for data generation were directly derived from the ESM data.

The results (see Figure S7 and S8) were consistent with the findings in the paper. Most models improved convergence, except for the type A models. For simplicity, we focused on the estimation of actor effect of females at both within- and between-person levels. The results

showed no notable biases in within-person effect. Interestingly, the estimation of the between-person effect also showed no significant biases, indicating that while the estimation bias of the between-person effect cannot be improved by alternative models, as stated in the paper, it is related to the true effect, which we will not explore further here. The SE of both within- and between-person effects were related to sample size and varied minimally across most alternative models.

Figure S7

Number of converged replicates across 1000 replicates (ESM data)

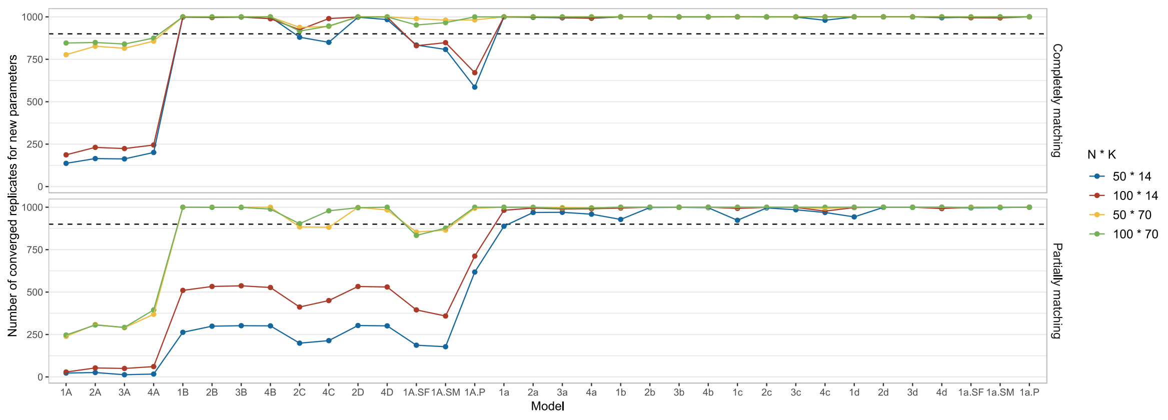

Figure S8

Relative bias and SE Number of converged replicates across 1000 replicates (ESM data)

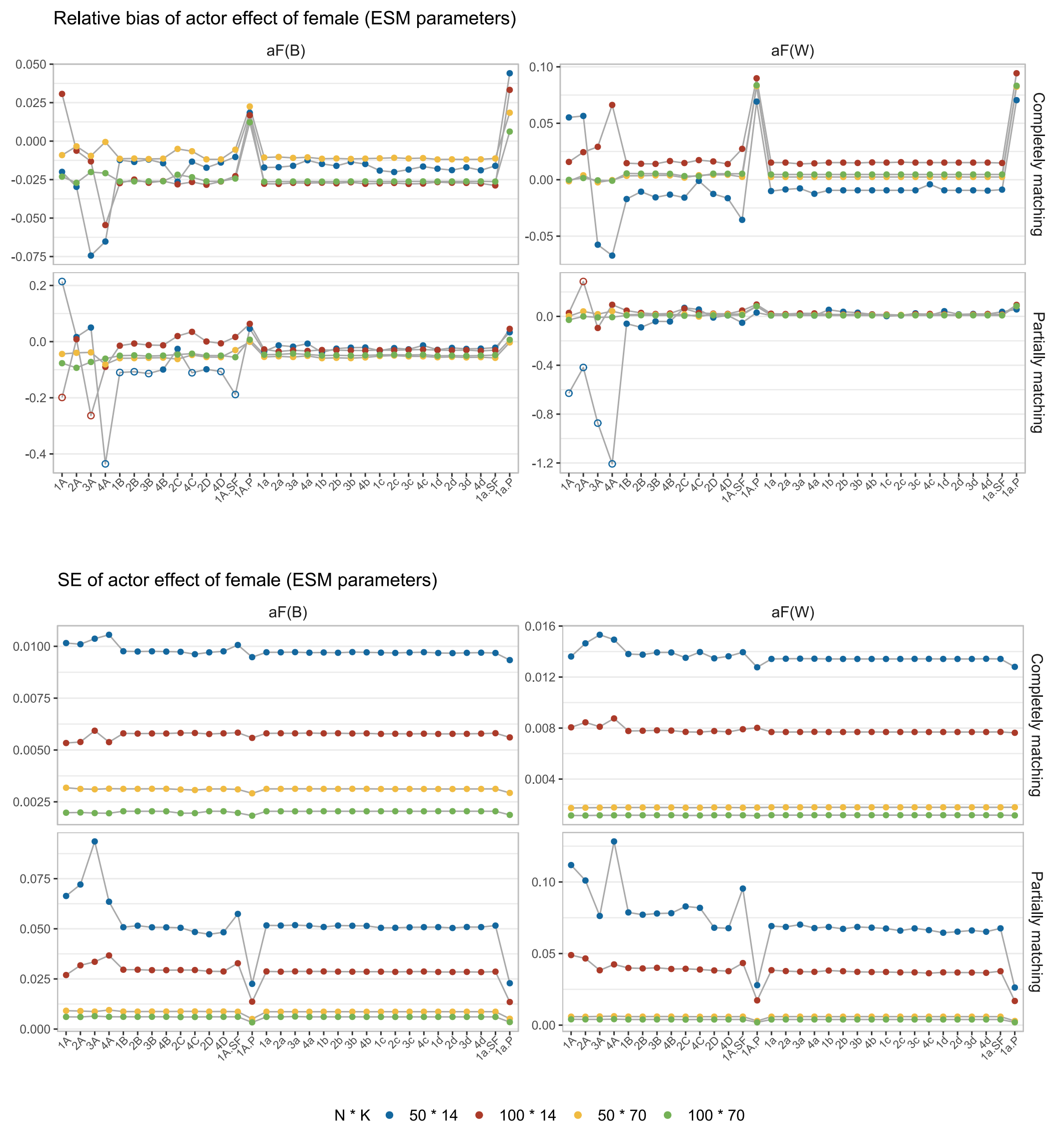

Supplement: Ji et al. supplementary material [file S0033312325000183sup001.pdf]
